# Unlocking the Potential of *N*,*N*,*N*′,*N*′‑Tetraphenylbenzidine Based on Conjugated Microporous Polymers for Rhodamine B Adsorption: A Synergistic Experimental and Density Functional Theory Perspective

**DOI:** 10.1021/acspolymersau.5c00025

**Published:** 2025-06-10

**Authors:** Mohammed G. Kotp, Mohamed Gamal Mohamed, Pei-Tzu Wang, Ahmed E. Hassan, Ahmed M. Elewa, Shiao-Wei Kuo

**Affiliations:** † Department of Materials and Optoelectronic Science, Center for Functional Polymers and Supramolecular Materials, 34874 National Sun Yat-Sen University, Kaohsiung 804, Taiwan; ‡ Chemistry Department, Faculty of Science, 68796 Assiut University, Assiut 71515, Egypt; § Interdisciplinary Research Center for Hydrogen Technologies and Carbon Management (IRC-HTCM), King Fahd University of Petroleum & Minerals, Dhahran 31261, Saudi Arabia; ∥ Department of Chemistry, Faculty of Science, 68820 Al-Azhar University, 11884 Nasr City, Cairo, Egypt; ⊥ Department of Chemical Engineering, 34881 National Tsing Hua University, Hsinchu 300044, Taiwan; # Department of Medicinal and Applied Chemistry, Kaohsiung Medical University, Kaohsiung 807, Taiwan

**Keywords:** *N*, *N*, *N*′, *N*′-tetraphenylbenzidine, conjugated microporous polymers, Thermal stability, rhodamine B, adsorption, water purification, Adsorption kinetics

## Abstract

This study presents the synthesis and construction of two innovative conjugated microporous polymers (CMPs), TPBZ-PyT CMP and TPBZ-TPET CMP, which incorporate pyrene (Py) and tetraphenylethene (TPE) subunits, respectively. These subunits are selected for their complementary properties, with the TPE moiety offering enhanced flexibility compared to the more rigid Py structure. The flexibility of TPE is hypothesized to improve the adsorption performance of the CMPs for removing rhodamine B (RhB) dye from aqueous solutions. The TPBZ CMPs were characterized using a suite of techniques, including Brunauer–Emmett–Teller (BET) surface area analysis, SEM, and FTIR. Batch adsorption experiments demonstrated that TPBZ-TPET CMP achieved a remarkable RhB removal efficiency of 49.49% within the first 30 min, reaching 98.72% after 60 min. In comparison, TPBZ-PyT CMP attained a maximum removal efficiency of 53.49% at the 60 min mark. Kinetic studies revealed distinct adsorption mechanisms for the two TPBZ CMPs. The adsorption process for TPBZ-TPET CMP was analyzed using a pseudo-second-order model, showing that chemisorption is the dominant mechanism. Meanwhile, TPBZ-PyT CMP exhibited pseudo-first-order kinetics, suggesting a different rate-limiting step. These findings highlight the critical role of subunit flexibility in designing CMPs for enhanced adsorption performance. The superior efficiency of TPBZ-TPET CMP underscores the potential of flexibility-engineered CMPs in advancing water purification technologies and addressing dye contamination in aquatic environments.

## Graphical abstract

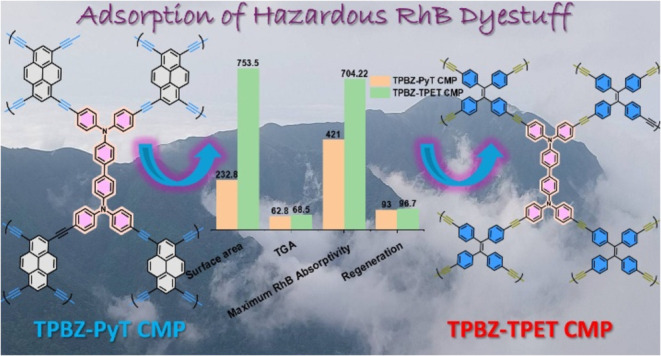

## Introduction

The presence of hazardous organic dyes in industrial wastewater poses a severe threat to aquatic ecosystems and human health due to their carcinogenic, mutagenic, and toxic properties.^1–5^ Among these dyes, rhodamine B (RhB) is particularly harmful, as it can cause respiratory and ocular irritation, neurotoxicity, and chronic health issues in humans and animals upon ingestion.^6–10^ The effective removal of RhB from water sources is critical to mitigating its detrimental effects.^11^ Various techniques have been developed to address this issue, with adsorption emerging as a widely adopted method due to its cost-effectiveness and environmentally friendly nature. Among the different adsorption approaches, polymeric materials have shown significant promise in efficiently removing organic dyes from contaminated water.^12,13^

Conjugated microporous polymers (CMPs) have attracted considerable interest as promising adsorbents owing to their superior surface area, tunable structures, and excellent thermal and chemical stability.^14–21^ The incorporation of electron-rich and redox-active building blocks, such as triphenylamine (TPA) derivatives, into CMPs can further enhance their adsorption performance for the removal of organic dyes.^22–24^ TPA-based CMPs, in particular, have demonstrated great potential as effective adsorbents due to their ability to interact with organic pollutants through π–π stacking and hydrogen bonding interactions.^25^ However, many existing porous adsorbents, despite their effectiveness, face challenges such as low adsorption capacities, poor reusability, and slow kinetics.^26–28^ To overcome these limitations, the development of novel CMPs focuses on exploiting their high surface area, tunable porosity, chemical robustness, and structural flexibility.^29^ These advancements aim to create CMPs capable of achieving superior adsorption performance, enhanced reusability, and faster adsorption kinetics, making them highly effective for addressing organic dye contamination in water.

Utilizing *N*,*N*,*N*′,*N*′-tetraphenylbenzidine (TPBZ) derivatives as subunits for constructing conjugated microporous polymers (CMPs) represents a promising strategy to enhance the adsorption capabilities of these materials for removing RhB and other pollutants from water. The incorporation of TPBZ into CMPs significantly improves their structural stability and increases the number of active adsorption sites due to TPBZ’s aromatic and electron-rich nature.^30^ This facilitates stronger π–π interactions and electrostatic attractions with cationic dyes like RhB, which are essential for efficient adsorption.^31^ Moreover, the inherent flexibility of the TPBZ subunit allows CMPs to accommodate larger dye molecules, thereby promoting higher adsorption capacities.^32,33^ The tunable nature of CMPs, achieved by modulating the linkage monomers, further enables the optimization of pore size and surface area, critical parameters that directly influence adsorption efficiency.^34^ By systematically designing CMPs with TPBZ subunits, researchers can improve the adsorption kinetics and the effectiveness of dye removal processes. This innovative approach not only enhances RhB removal but also broadens the potential applications of these CMPs to a wider range of organic pollutants. Such advancements contribute to the development of sustainable and effective water purification technologies, addressing the pressing challenges of water contamination. Thus, the novelty of this work lies in the systematic comparison of rigid versus flexible subunits in conjugated microporous polymers, establishing for the first time a direct correlation between subunit flexibility and enhanced adsorption performance for hazardous dyes.

In this study, we designed and synthesized two CMPs, TPBZ-PyT CMP and TPBZ-TPET CMP, incorporating pyrene (Py) and tetraphenylethene (TPE) subunits, respectively, along with a fixed TPBZ unit, as presented in Figure 1. These CMPs were developed to evaluate their adsorption performance for the removal of RhB dye. Incorporating Py and TPE aimed to enhance the flexibility and adsorption capacity of the CMPs. To the best of our knowledge, the impact of subunit flexibility on CMP adsorption performance has not been previously explored. The planar structure of Py was expected to promote π–π stacking interactions with RhB molecules, while the bulky and flexible TPE moiety was anticipated to facilitate the accommodation of RhB through a more favorable structural environment.^35,36^ Specifically, the use of TPE in TPBZ-TPET CMP was hypothesized to enhance adsorption capacity compared to the more rigid Py subunits in TPBZ-PyT CMP due to its greater structural flexibility. This study provides a direct comparison of the adsorption performance of TPBZ-PyT CMP and TPBZ-TPET CMP, allowing us to evaluate the role of subunit flexibility in RhB dye removal efficiency. Furthermore, adsorption kinetics and mechanisms were thoroughly investigated using pseudo-first-order, pseudo-second-order, and intraparticle diffusion models. These analyses offered insights into the rate-controlling steps and the interactions between the CMPs and RhB dye, establishing a clear correlation between the structural properties of the CMPs and their adsorption efficiency. The findings of this study contribute to the development of advanced CMP-based adsorbents for efficient water purification, addressing the critical issue of dye contamination in aquatic environments.

**Figure 1:**
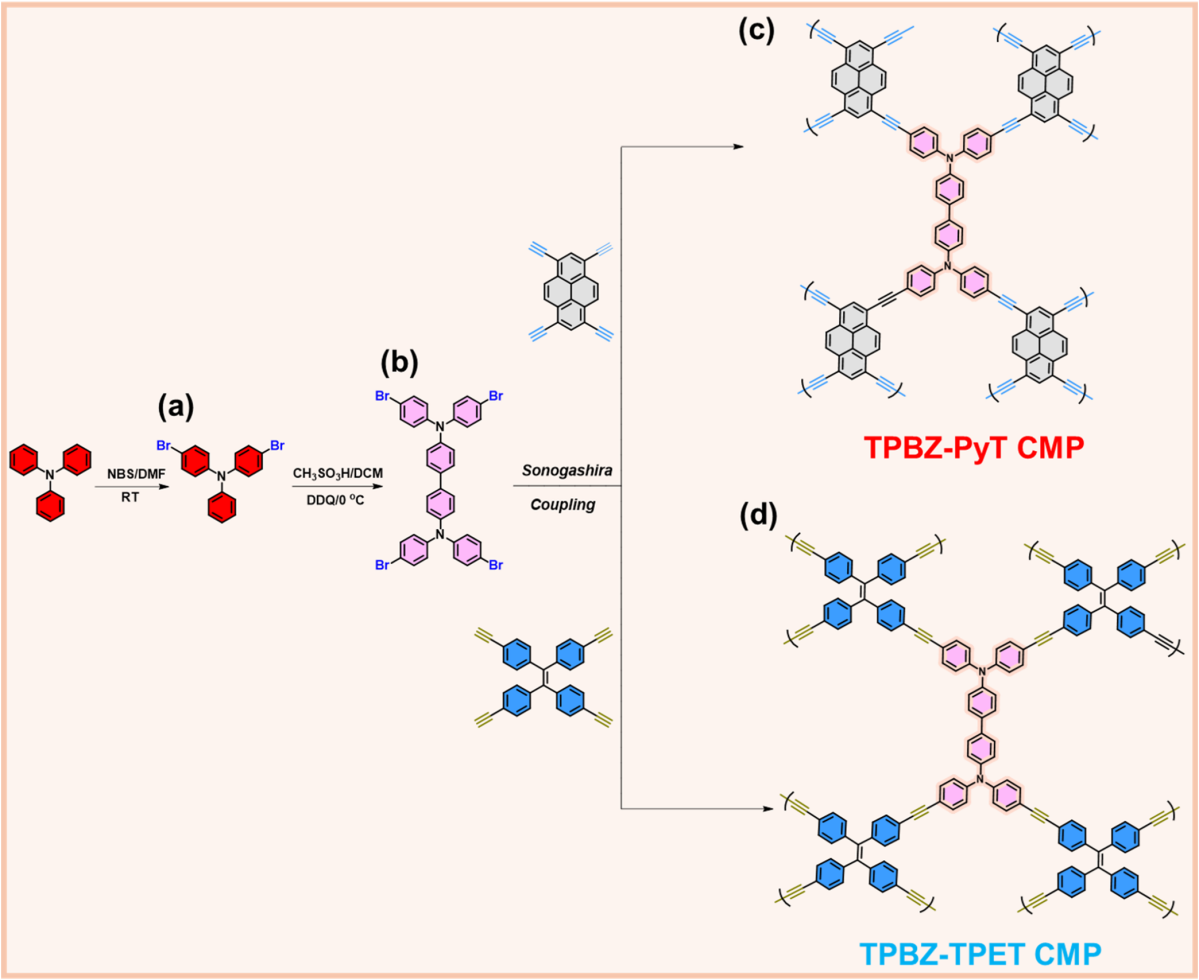
Schematic representation for the synthesis of (a) TPA-Br_2_, (b) TPBA-Br_4_, (c) TPBZ-PyT CMP, and (d) TPBZ-TPET CMP.

In this study, TPBZ-PyT and TPBZ-TPET CMPs were thoroughly characterized using a range of advanced techniques, including FTIR, SS ^13^C NMR, XPS, Brunauer–Emmett–Teller (BET) surface area analysis, SEM, and TEM. TPBZ-TPET CMP demonstrated large surface areas reaching up to 754 m^2^ g^–1^, along with smooth, spherical-like morphologies. The adsorption characteristics of RhB dye on these TPBZ CMPs. as evaluated through batch adsorption experiments. Kinetic studies and adsorption mechanisms were investigated using pseudo-first-order, pseudo-second-order, and intraparticle diffusion models. Notably, the flexible TPBZ-based CMPs demonstrated superior adsorption capacities, achieving RhB removal of up to 704 mg g^–1^. The findings emphasize the pivotal influence of subunit flexibility in improving the adsorption performance of CMPs. This study not only underscores the potential of flexible CMPs for effective RhB removal but also contributes to the broader development of advanced adsorbent materials for water purification. These findings offer valuable insights into addressing the pressing issue of dye contamination in aquatic environments and pave the way for sustainable solutions to water pollution challenges.

## Experimental Section

### Materials

Triphenylamine (TPA), copper­(I) iodide (CuI, 99%), potassium carbonate (K_2_CO_3_), methanesulfonic acid (CH_3_SO_3_H), N-bromosuccinimide (NBS), trimethylsilylacetylene (TMSA), dimethylformamide (DMF), dichloromethane (DCM), tetrakis­(triphenylphosphine)­palladium(0) [Pd­(PPh_3_)_4_], methanol (MeOH), 2,3-dichloro-5,6-dicyanobenzoquinone (DDQ), and triethylamine (Et_3_N) were procured from Sigma-Aldrich and Acros. The detailed preparation of Py and TPE-functionalized alkynyl groups [PyT and TPET] via the Sonogashira reaction is provided in the Supporting Information file, as well as their FTIR and NMR results [Figures S1–S6].^37–41^

### Synthesis of N4,N4,N4′,N4′-tetrakis­(4-bromophenyl)-[1,1′-biphenyl]-4,4′-diamine [TPBZ-Br_4_]

The synthesis of the TPBZ monomer was carried out following the reported protocol.^42^ In a 140 mL flask, 10.00 g (40.8 mmol) of TPA was dissolved in 80 mL of DMF. To this solution, 14.41 g (81.0 mmol) of NBS was added, followed by an additional 80 mL of DMF and heated to 70 °C for 24 h. The resulting solid was isolated from the solvent using vacuum distillation. The solid product was purified via column chromatography, yielding an ivory-white viscous solid identified as 4-bromo-N-(4-bromophenyl)-*N*-phenylaniline (TPA-Br_2_). ^1^H NMR [Figure S7a]: 7.4 (4H), 7.3 (2H), 7.1 (3H), 6.94 (4H). ^13^C NMR [Figure S7b]: 115.4–147.33. For the next step, 4.81 g of TPA-Br_2_ was dissolved in 300 mL of DCM and cooled in an ice bath until the temperature reached 0 °C. After maintaining this temperature for 5 min, 45.09 g (469.10 mmol) of CH_3_SO_3_H and 4.50 g (19.8 mmol) of DDQ were added to the mixture. The product was then extracted using DCM and water. To remove any residual water, magnesium sulfate was added, followed by filtration. The DCM solvent was evaporated, and the product was recrystallized from acetonitrile, yielding a white powder of the TPBZ-Br_4_ monomer. FTIR (Figure S8): 3031 (Ar–H), 1600 (C=C) cm^–1^. ^1^H NMR [Figure S9a]: 7.5 (2H), 7.4 (4H), 7.11 (2H), 6.99 (4H). ^13^C NMR [Figure S9b]: 146.34–115.7.

### Synthesis of TPBZ-PyT CMP and TPBZ-TPET CMP

Utilizing the simple Sonogashira protocol,^43^ and under a nitrogen atmosphere, a mixture of 0.15 g (0.183 mmol) TPBZ-Br_4_, 0.055 g (0.183 mmol) of PyT or 0.08 g (0.183 mmol) of TPET, 0.05 g (0.26 mmol) of CuI, 0.05 g (0.17 mmol) of PPh_3_, and 0.06 g (0.052 mmol) of Pd­(PPh_3_)_4_ was prepared in a Schlenk flask. To this, 15 mL each of DMF and Et_3_N were added as solvents. The mixture was heated at 110 °C for 72 h. After the reaction was complete, the precipitate was separated through filtration and extensively washed with THF, methanol, and acetone using Soxhlet extraction. This procedure produced TPBZ-PyT CMP as a dark red powder with a yield of 90%. This powder was then suspended in methanol for 1 h, followed by filtration to obtain a clear and deep red solid and a dark yellow powder with a 95% yield for TPBZ-TPET CMP.

### RhB Dyestuff Adsorption

RhB dye, a widely used cationic dye, serves as a luminous water flow indicator and is commonly applied to evaluate the adsorbent properties of materials.^44^ Due to its ability to be easily monitored using a standard UV–visible spectrum analyzer, RhB was selected to study the adsorption capacity of the synthesized TPBZ-based CMPs. In the experiment, 5 mg of TPBZ-PyT CMP and TPBZ-TPET CMP were added to 10 mL of an RhB solution (25 mg L^–1^) in a 20 mL tube. Notably, the concentration of RhB used in this study was deliberately selected to ensure detectable adsorption behavior and to enable a thorough evaluation of the adsorption performance of our synthesized TPBZ-PyT and TPBZ-TPET CMPs. Although RhB concentrations in natural environments are generally lower, elevated concentrations are commonly employed in laboratory-scale experiments to simulate worst-case scenarios, test adsorbent capacity under demanding conditions, and gain deeper insights into adsorption mechanisms. This approach helps establish a benchmark for the maximum adsorption efficiency and kinetic behavior of the materials, which is essential for guiding their design and practical implementation. Realistically, the mixture was stirred at 400 rpm under moderate magnetic stirring at pH 7 and 298 K. In our earlier study,^45^ we observed that the deprotonation of carboxylic groups in RhB molecules at pH 4–7 promotes the formation of dye dimers involving carboxylate ions and xanthene groups. This phenomenon slightly enhances adsorption efficiency in this pH range compared to basic conditions. As a result, a pH of 7 was selected as the ideal condition for the adsorption experiments. To monitor the adsorption process, the concentration of RhB in the solution was measured at various time intervals. RhB molecules were separated from the aqueous media using centrifugation at 5000 rpm. Adsorption kinetics and isotherms were studied by adjusting the initial RhB concentrations between 25 and 200 mg L^–1^. For these experiments, 10 mL of RhB solution at a specific concentration was mixed with 2 mg of TPBZ CMPs and allowed to equilibrate for 24 h. The adsorption kinetics and equilibrium isotherms were analyzed using the Langmuir (eq 1) and Freundlich (eq 2) models to elucidate the adsorption mechanisms and capacities. Additionally, the potential for reusing TPBZ CMPs as dye adsorbents was examined. The adsorbents were regenerated using a combination of acetone and water between cycles, and their performance was evaluated under identical adsorption conditions.

1 $$\frac{C_{e}}{Q_{e}}=\frac{1}{K_{L}Q_{m}}+\frac{C_{e}}{Q_{m}}$$

2 $$\ln Q_{e}=\ln K_{F}+\frac{1}{n}\ln C_{e}$$

In which *Q*_e_ (mg g^–1^) is the equilibrium capacity for adsorption, *Q*_m_ is the ultimate capacity. The *K*_L_ and *K*_F_ are the coefficients of Langmuir and Freundlich respectively, where *n* is the sorption amplitude. *C*_e_ (mg L^–1^) is the dye concentration after equilibrium.

## Results and Discussion

### Characterization of TPBZ-Br_4_, TPBZ-PyT CMP, and TPBZ-TPET CMP

As illustrated in Figure 1, the synthesis of the primary building block, TPBZ-Br_4_, involves two sequential steps. First, TPA is reacted with NBS in the presence of DMF to produce TPA-Br_2_ [Figure 1a]. In the second step, TPA-Br_2_ undergoes a reaction with CH_3_SO_3_H in DCM and DDQ at 0 °C, yielding TPBZ-Br_4_ as a white solid. To synthesize TPBZ-PyT CMP and TPBZ-TPET CMP [Figure 1c,d], TPBZ-Br_4_ is reacted with PyT and TPET, respectively, via a Sonogashira coupling reaction, resulting in the desired TPBZ CMPs. To confirm the structures of the monomers, including PyT, TPET, and TPBZ-Br_4_, we employed NMR and FTIR spectroscopy. The FTIR spectrum of the PyT monomer [Figure S1] exhibits characteristic absorption bands at 2186 cm^–1^ (C≡C stretching) and 3279 cm^–1^ (alkyne C–H stretching). The ^1^H NMR spectrum of PyT [Figure S2] reveals a signal at 3.66 ppm corresponding to protons adjacent to the triple bond, alongside signals at 8.67 and 8.34 ppm, which are attributed to aromatic protons. The ^13^C NMR spectrum of PyT [Figure S3] displays signals at 81.62 and 84.13 ppm, corresponding to the triple bonds, and four peaks in the range of 127.72–132.57 ppm, confirming the presence of aromatic carbons. For the TPET monomer, the FTIR spectrum [Figure S4] shows a C=C stretching band at 1617 cm^–1^, a weak C≡C stretching band at 2109 cm^–1^, an aromatic C–H stretching band at 3042 cm^–1^, and an alkyne C–H stretching band at 3273 cm^–1^, confirming the coexistence of benzene rings and alkyne groups. The ^1^H NMR spectrum of TPET [Figure S5] features signals at 3.07, 7.29, and 6.95 ppm, indicating the presence of triple bonds and aromatic rings. The ^13^C NMR spectrum of TPET [Figure S6] shows characteristic peaks for triple bonds at approximately 78.28 and 83.77 ppm, along with signals for aryl carbons at 120.99, 132.54, 141.50, and 143.88 ppm. The FTIR spectrum of the TPBZ-Br_4_ [Figure S8] displays absorption bands at 3060 cm^–1^, associated with C–H stretching vibrations in aromatic groups. In the ^1^H NMR spectrum [Figure S9a], sharp peaks in the range of 7.1–7.6 ppm indicate the presence of aryl protons (Ar–H). Additionally, the ^13^C NMR spectrum shows chemical shifts between 110–150 ppm, confirming the aromatic carbon sites [Figure S9b]. The molecular characterization of the two TPBZ CMPs was conducted using FTIR, solid-state ^13^C NMR spectroscopy (SS ^13^C NMR), and XPS. The FTIR spectra exhibited distinct peaks within the range of 2890–3004 cm^–1^, corresponding to aromatic C–H vibrations, and 2127–2255 cm^–1^, indicative of C≡C units.

Additionally, absorption bands between 1673 and 1553 cm^–1^ were assigned to aromatic C=N and C=C stretching vibrations [Figure 2a]. The solid-state ^13^C NMR spectrum, shown in Figure 2b, displayed signals in the regions of 60–102 ppm and 109–156 ppm, representing two primary types of carbons within the networks: aromatic carbons and those associated with C≡C units. These findings confirm the structural integrity of the CMPs. XPS analysis was done on TPBZ-PyT and TPBZ-TPET CMPs to examine their elemental composition and the chemical states of key functional groups [Figure S10]. The obtained data, including binding energies (B.E.), full width at half-maximum (fwhm), and peak areas for carbon (C) and nitrogen (N), are summarized in Table S1. These parameters provide valuable insights into the structural characteristics of the TPBZ CMPs, which are crucial for their effectiveness as adsorbents in dye removal applications. The C 1s peaks [Figure 3a] provide detailed information about the carbon bonding environments in the CMPs. A prominent peak at a binding energy of 283.85 eV, corresponding to C=C bonding, is observed in both TPBZ-PyT and TPBZ-TPET CMPs. This confirms the conjugated nature of these polymers, a feature essential for their electronic properties and their interaction with organic pollutants such as RhB. Additionally, the C–N bonding peak at 284.85 eV [Figure 3a] indicates the successful incorporation of nitrogen-containing groups into the TPBZ CMPs, contributing significantly to their adsorption properties. The area under this peak is notably larger for TPBZ-PyT CMP compared to TPBZ-TPET CMP, suggesting a higher abundance of nitrogen functionalities in the former. This increased nitrogen content may enhance the dye adsorption capabilities of TPBZ-PyT CMP. The presence of C–O bonding, represented by a peak at 285.33 eV, suggests the inclusion of hydroxyl or ether groups in both CMPs. These functionalities facilitate interactions with adsorbates via hydrogen bonding or dipole–dipole interactions, further improving the adsorption capacity of the materials. The N 1s peaks [Figure 3b] offer valuable insights into the nitrogen content and its bonding environment within the CMPs. A prominent peak at a binding energy of 398.91 eV, corresponding to N–C bonding, is observed in both TPBZ-PyT and TPBZ-TPET CMPs. This indicates that nitrogen atoms are effectively bonded to carbon, contributing to the structural integrity of the CMPs. Notably, the area under the N–C peak is significantly larger for TPBZ-PyT CMP compared to TPBZ-TPET CMP, suggesting a higher nitrogen content in TPBZ-PyT CMP. This increased nitrogen content enhances the material’s potential for interactions with cationic dyes such as RhB, further reinforcing its effectiveness as an adsorbent.

**Figure 2:**
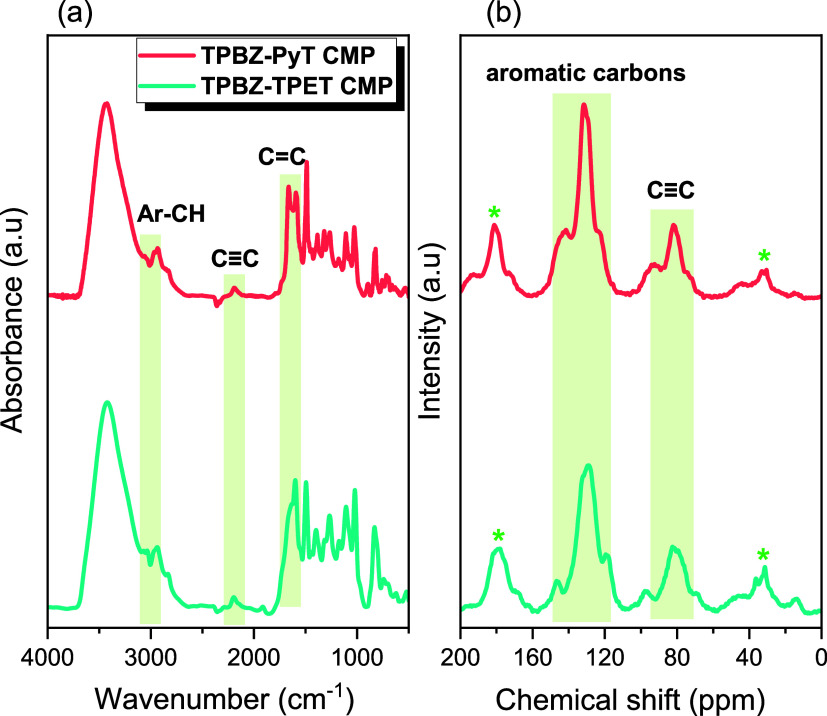
(a) FTIR and (b) SS ^13^C NMR of TPBZ-PyT and TPBZ-TPET CMPs.

**Figure 3:**
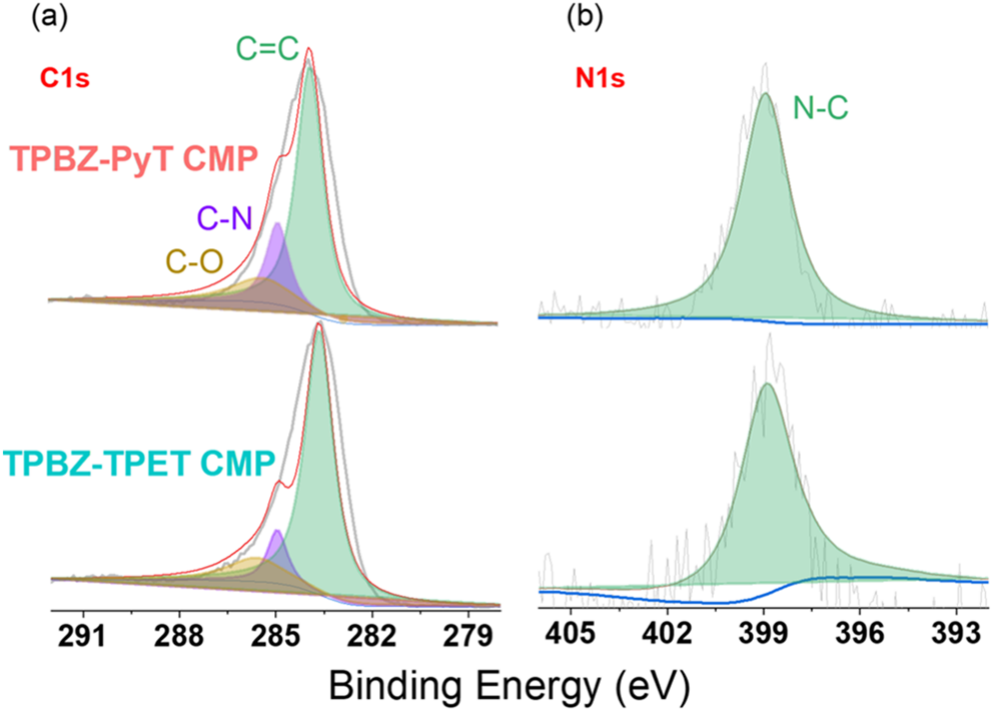
HRXPS of (a) C and (b) N incorporated TPBZ-PyT and TPBZ-TPET CMPs.

Notably, the TPBZ-PyT and TPBZ-TPET CMPs exhibit insolubility in common organic solvents such as methanol, acetone, and THF, enabling efficient separation from the reaction solvent after synthesis. This insolubility is a critical feature for practical applications and processing, as it ensures the isolation of pure TPBZ CMP products without contamination from residual solvents or unreacted monomers.^46^ The insolubility of these TPBZ CMPs is attributed to their highly cross-linked structure, resulting from polymerization reactions that produce extensive networks with restricted molecular mobility. These robust frameworks are further stabilized by strong intermolecular interactions, including π–π stacking and hydrogen bonding, which hinder solvation by organic solvents. This property offers significant advantages in applications such as catalysis and sensing, where separating the solid CMPs from liquid phases is essential. Additionally, the insolubility of TPBZ-PyT and TPBZ-TPET CMPs suggests unique structural features that enhance their functional versatility. The inability to dissolve in conventional solvents indicates a durable framework capable of withstanding harsh environmental conditions, making these materials well-suited for demanding applications such as environmental remediation and chemical sensing.^47,48^

In this study, TGA was utilized to analyze the thermal stability of two CMPs, TPBZ-PyT and TPBZ-TPET, under a nitrogen atmosphere [Figure 4]. The analysis revealed char yields of 63 wt % for TPBZ-PyT CMP and 69 wt % for TPBZ-TPET CMP, indicating that both materials exhibit excellent thermal resistance, comparable to previously reported values. The char yield reflects the percentage of the original mass remaining after thermal decomposition, serving as a key indicator of the material’s ability to endure high temperatures without significant degradation. A higher char yield is typically associated with stronger intermolecular interactions and a more robust polymer structure, which enhances the material’s ability to retain its integrity under elevated temperatures. The degradation temperatures corresponding to a 10 wt % mass loss were determined to be 361 °C for TPBZ-PyT CMP and 497 °C for TPBZ-TPET CMP. These temperatures are critical indicators of thermal stability, marking the onset of significant decomposition processes. The notable difference in degradation temperatures indicates that TPBZ-TPET CMP demonstrates enhanced thermal stability in comparison to TPBZ-PyT CMP. This enhanced stability is likely due to TPBZ-TPET’s higher cross-linking density, which strengthens its structural integrity and resistance to thermal degradation.^49^

**Figure 4:**
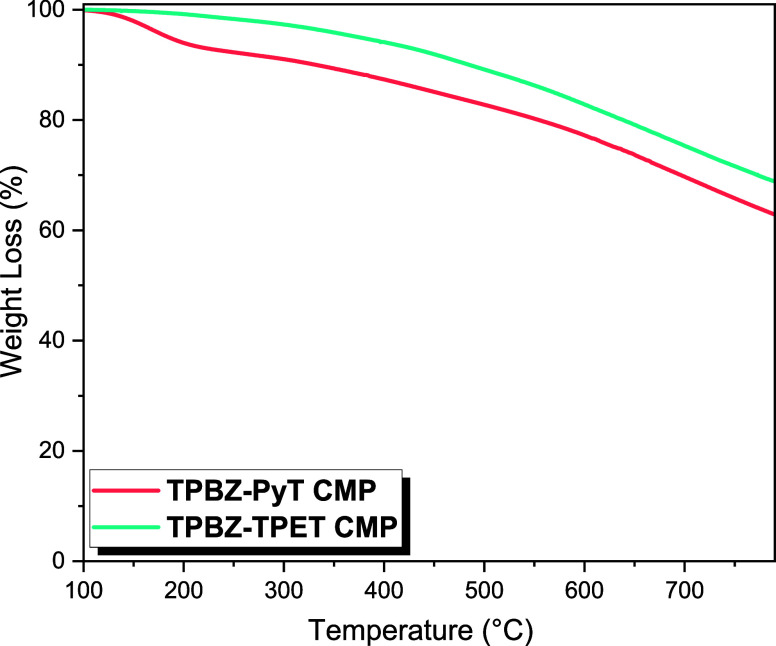
TGA profiles of TPBZ-PyT and TPBZ-TPET CMPs.

To assess the porosity characteristics of TPBZ-PyT and TPBZ-TPET CMPs, N_2_ adsorption/desorption measurements were conducted at 77 K. The results reveal that both TPBZ CMPs exhibit adsorption isotherms classified as a combination of Type II and Type IV, according to IUPAC standards [Figure 5a]. This classification provides valuable insights into the pore structures and adsorption behavior of the materials. Type II isotherms are generally linked to nonporous or macroporous materials, indicating unrestricted mono- and multilayer adsorption at elevated relative pressures.^50^ In contrast, Type IV isotherms are indicative of mesoporous materials, characterized by the presence of hysteresis loops that signify multilayer adsorption on mesoporous surfaces, with pore sizes ranging from 0.27 to 50 nm.^51^ The combination of these isotherm types in TPBZ-PyT and TPBZ-TPET CMPs suggests a complex pore structure comprising both mesopores and micropores, which enhances their potential for adsorption and separation applications. The observed hysteresis loops in the N_2_ adsorption/desorption isotherms [Figure 5a] further indicate that the TPBZ CMPs possess a framework structure capable of retaining N_2_ gas within their pores during desorption. This behavior is characteristic of materials with interconnected mesoporous and microporous architectures, particularly those with narrow pore sizes. The hysteresis can be attributed to the filling and emptying of pores during the adsorption and desorption processes, influenced by factors such as pore connectivity and size distribution. This intricate pore structure enhances the functionality of the CMPs in applications requiring efficient adsorption and separation. TPBZ-PyT CMP had a BET surface area of 233 m^2^ g^–1^ and a pore volume of 0.2584 cm^3^ g^–1^, while TPBZ-TPET CMP exhibited much higher values of 754 m^2^ g^–1^ and 0.7383 cm^3^ g^–1^. These results underscore the enhanced porosity of TPBZ-TPET CMP, which suggests superior performance in applications requiring extensive surface area interactions, such as catalysis, adsorption, or gas storage. The pyrene subunit in TPBZ-PyT CMP is a rigid, planar aromatic structure that promotes ordered stacking during polymerization. This intrinsic rigidity often leads to dense molecular packing and limited pore accessibility, thereby reducing the overall surface area. In contrast, the tetraphenylethene (TPE) subunit in TPBZ-TPET CMP introduces significant structural flexibility due to its nonplanar, twisted conformation, which arises from steric hindrance among the peripheral phenyl rings. This flexibility disrupts tight packing and facilitates the formation of a more disordered and porous framework with enhanced microporosity. The nonplanar geometry of the TPE unit promotes the generation of interconnected micropores during the Suzuki coupling reaction, resulting in a higher degree of cross-linking and a more open network architecture. As a consequence, TPBZ-TPET CMP exhibits a substantially increased BET surface area. In contrast, the planar nature of the pyrene moiety favors linear or sheet-like polymer growth, which tends to yield more compact and less porous structures. These structural distinctions highlight how subunit design directly governs the porosity and surface properties of CMPs. The pore size distribution of the TPBZ CMPs was analyzed using nonlocal density functional theory (NLDFT), revealing distinct characteristics for each material. TPBZ-PyT CMP exhibited a primary peak at 1.70 nm, with additional peaks observed between 3.75 and 6.72 nm [Figure 5b], indicative of a range of narrow pores well-suited for selective adsorption processes. In comparison, TPBZ-TPET CMP displayed a main peak at 1.40 nm, along with a smaller peak at 0.27 nm, and additional peaks ranging from 3.60 to 6.71 nm [Figure 5b]. This broader distribution of pore sizes suggests that TPBZ-TPET CMP possesses greater versatility, enabling it to accommodate a wider variety of guest molecules compared to TPBZ-PyT CMP. The structural analysis of TPBZ-PyT and TPBZ-TPET CMPs was performed using powder X-ray diffraction (XRD), SEM, and TEM, providing detailed insights into their physical properties. XRD was employed to assess the crystallinity of the materials, with the results confirming their amorphous nature [Figure S11]. This conclusion is supported by the absence of distinct sharp peaks in the diffraction patterns, a characteristic feature of amorphous materials. Amorphous structures lack long-range order, which can be advantageous in applications such as catalysis and gas adsorption, where increased surface area and enhanced accessibility to active sites are critical.^52^ SEM provides high-resolution images of the surface morphology of materials. The SEM images revealed that TPBZ-PyT CMP [Figure 5c,d] consists of irregularly aggregated spheres, indicating a diverse microstructure that may enhance its functional properties. Also, the TPBZ-TPET CMP was characterized by aggregated spheres [Figure 5e,f]. The SEM-EDS images supported the XPS data in Figure S10, confirming the existence of C and N atoms in the TPEBZ-TPET CMP and TPBZ-PyT CMP frameworks [Figures 5g,h and S12]. Based on SEM-EDS mapping, the atomic contents of C and N were 62.41 and 37.59%, respectively, for TPEBZ-TPET CMP, and 61.63 and 36.37% for TPBZ-PyT CMP. TEM offers insights into the internal structure at a nanoscale level, confirming the porous nature of TPBZ-PyT and TPBZ-TPET CMPs [Figure 5i,j], respectively, providing detailed insights into their physical properties.

**Figure 5:**
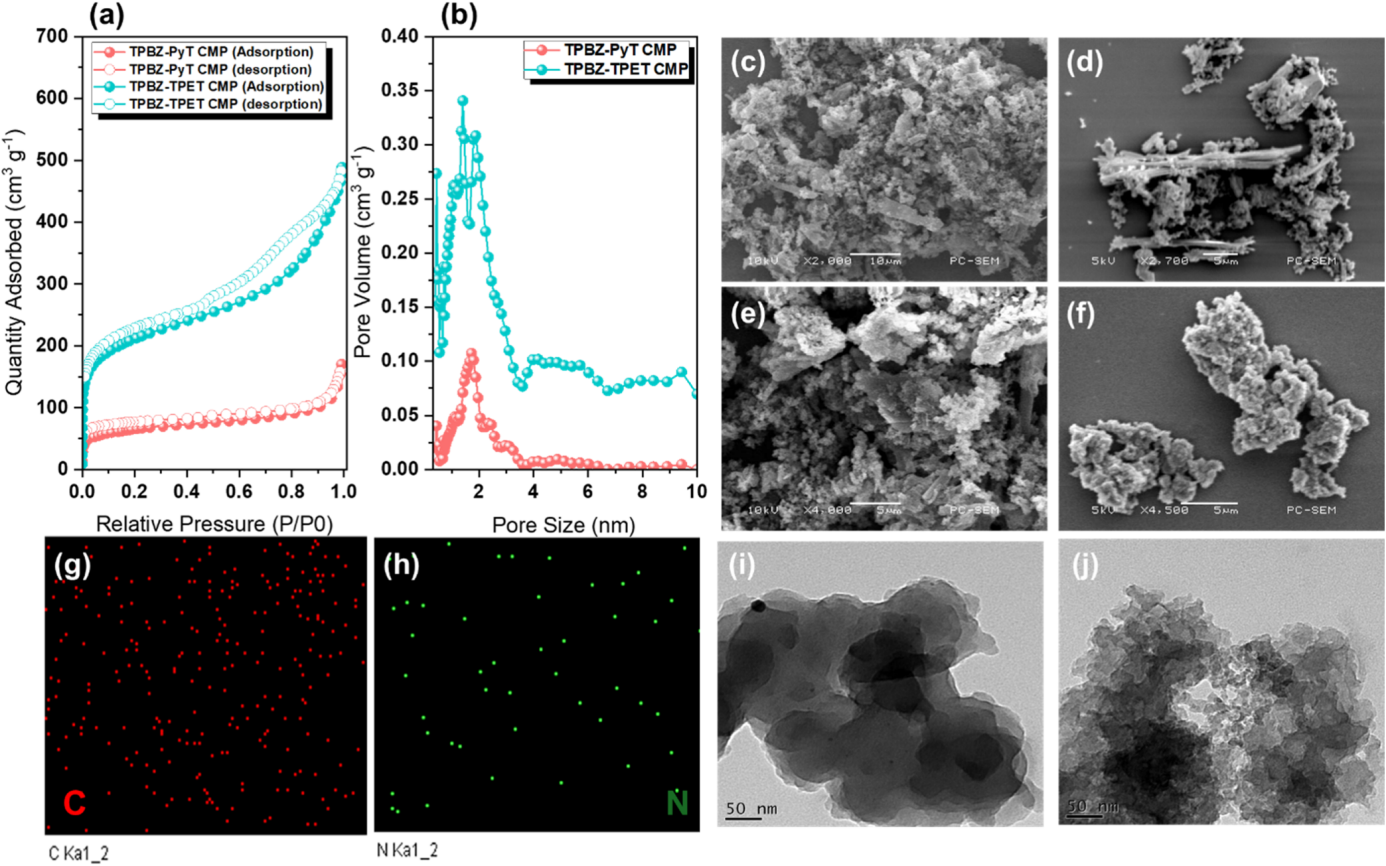
(a) N_2_ adsorption–desorption isotherm at 77 K and (b) pore size distribution of TPBZ CMPs. (c–f) SEM images of (c, d) TPBZ-PyT and (e, f) TPBZ-TPET CMPs. (g, h) SEM-EDS images of TPBZ-TPET CMP. (i, j) TEM images of (i) TPBZ-PyT and (j) TPBZ-TPET CMPs.

### RhB Adsorption Performance for TPBZ-PyT and TPBZ-TPET CMPs

The adsorption of RhB from aqueous solutions is governed by various intermolecular forces and the properties of the adsorbent.^53^ The aromatic structure of RhB enables π–π stacking interactions with adsorbents containing aromatic moieties, such as the CMPs discussed in this study. These face-to-face or edge-to-face interactions significantly enhance the adsorption capacity by facilitating close packing between the adsorbent and the dye molecules. Additionally, the adsorption process is heavily influenced by hydrogen bonding. The amino groups and oxygen atoms in RhB can form hydrogen bonds with hydrogen-donating or accepting chemical groups located on the surface of the adsorbent, further contributing to adsorption efficiency. Electrostatic interactions also have a significant influence on RhB adsorption, particularly as the process is strongly pH-dependent. At acidic pH, the positively charged RhB molecules interact electrostatically with negatively charged adsorbent surfaces, enhancing the adsorption process. This combination of π–π stacking, electrostatic interactions, and hydrogen bonding ensures effective adsorption of RhB under optimized conditions. The adsorbent’s characteristics, such as surface area and porosity, are essential in influencing its adsorption capacity. Adsorbents with larger surface areas and well-developed porous structures offer more adsorption sites, thereby improving the removal efficiency of RhB from aqueous solutions. The sorption capability of the evaluation of TPBZ CMPs was conducted using UV–visible spectroscopy by monitoring the effluent over a time window of 0–240 min. The characteristic UV-peak of RhB in aqueous solution at 554 nm [Figure S13] provided a simple and effective method to assess the adsorption performance of the TPBZ CMPs. Our TPBZ CMPs are nitrogen-functionalized materials that exhibit exceptional porosity, with pore volumes of 2.26 cm^3^ g^–1^ and significant surface areas reaching 754 m^2^ g^–1^. These unique properties suggest that TPBZ CMPs have strong potential as effective organic adsorbents. To evaluate their adsorption capacity, we investigated the performance of TPBZ-PyT CMP and TPBZ-TPET CMP against RhB, a widely recognized cationic dye molecule. RhB serves as both a fluorescent tracer and a benchmark compound for assessing adsorbent activity.^54^ As discussed earlier, the solution’s pH significantly influences the adsorption of RhB molecules. To explore this effect, control adsorption trials were conducted across a pH range of 1 to 12 [Figure 6a]. The results confirmed that elevated pH levels reduce the efficiency of RhB adsorption onto TPBZ CMPs. Limited adsorption under alkaline conditions was attributed to the transformation of RhB into its zwitterionic form (RhB±), which increases electrostatic repulsion and reduces interaction with the adsorbent. Conversely, the adsorption of RhB was significantly higher under acidic and neutral conditions [Figure 6a]. The zeta potential measurements for TPBZ-PyT and TPBZ-TPET CMPs were conducted over a wide pH range (1.0 to 12.0), providing valuable insights into the electrostatic interactions influencing the adsorption of RhB [Figure 6b]. The results revealed zero points of charge (ZPC) at approximately pH 7.01 for TPBZ-PyT CMP and pH 7.26 for TPBZ-TPET CMP. Below their ZPCs, in acidic conditions, both TPBZ CMPs exhibited a negative surface charge, which facilitated the attraction of positively charged RhB molecules. The adsorption capacity of the CMPs was significantly enhanced by this electrostatic interaction in both acidic and neutral environments, where protonation of amine groups on the CMPs further strengthened their interaction with RhB. In contrast, at pH levels above the ZPC, the surface charge of the CMPs became increasingly positive.

**Figure 6:**
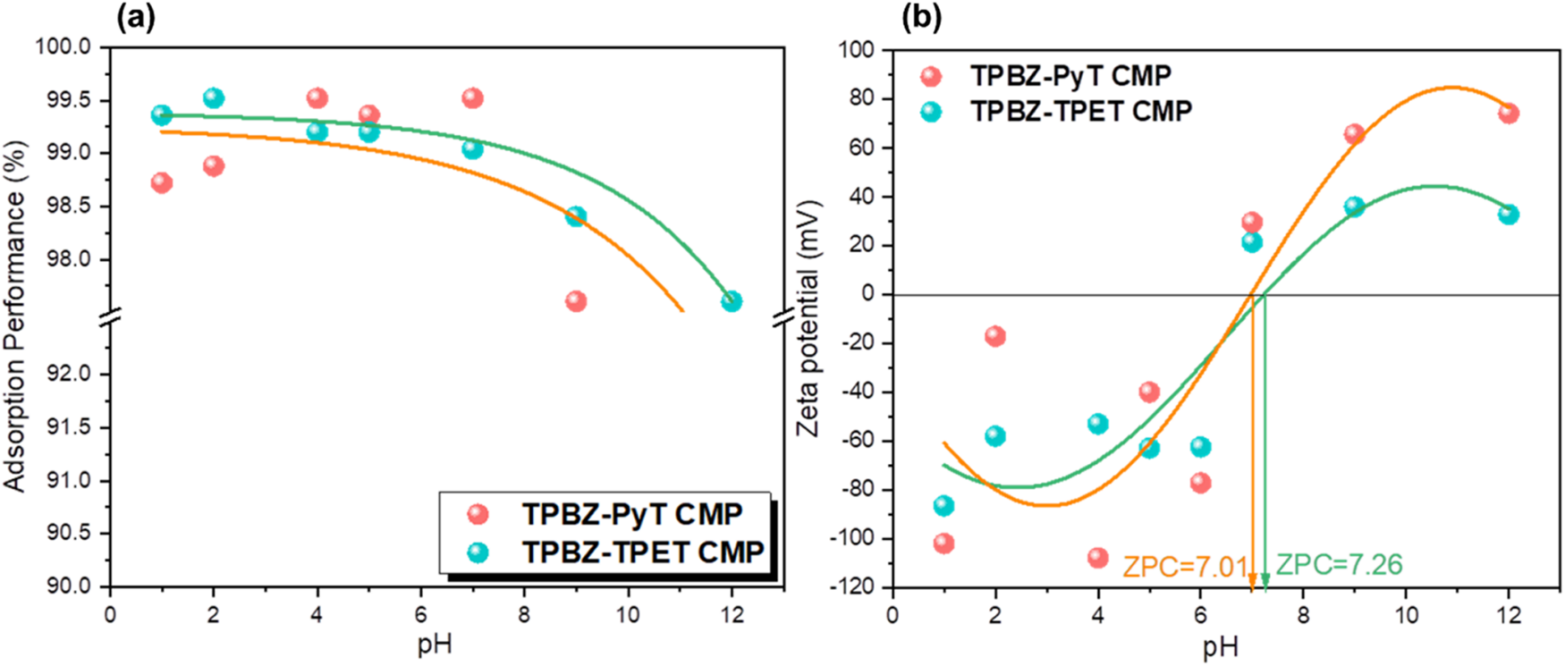
Adsorption of RhB dyestuff onto TPBZ-PyT and TPBZ-TPET CMPs and (b) zeta potential of TPBZ-PyT and TPBZ-TPET CMPs at various pHs.

This change resulted in electrostatic repulsion among the positively charged surfaces of TPBZ CMPs and the zwitterionic form of RhB, which predominates at higher pH levels. Consequently, the adsorption efficiency of RhB was significantly reduced under alkaline conditions. The equivalent concentration ratios (*C_t_*/*C*_0_) reveal a progressive removal rate of RhB, with TPBZ-TPET CMP demonstrating significantly faster adsorption efficiency compared to TPBZ-PyT CMP [Figure 7a]. The characteristic RhB absorption band at 554 nm steadily decreased in intensity, illustrating the adsorption process. Using TPBZ-TPET CMP, RhB removal reached 41% within the first 5 min and 63% within 10 min, with complete decolorization of the dye achieved in just 10 min [Figure 7b]. In contrast, the addition of 5 mg of TPBZ-PyT CMP to the aqueous RhB solution resulted in a slower removal rate, achieving 9 and 13% RhB removal in the first 5 and 10 min, respectively. Complete decolorization was observed only after 120 min [Figure 7b]. The significant difference in adsorption performance between the CMPs can be attributed to their distinct structural and chemical properties.

**Figure 7:**
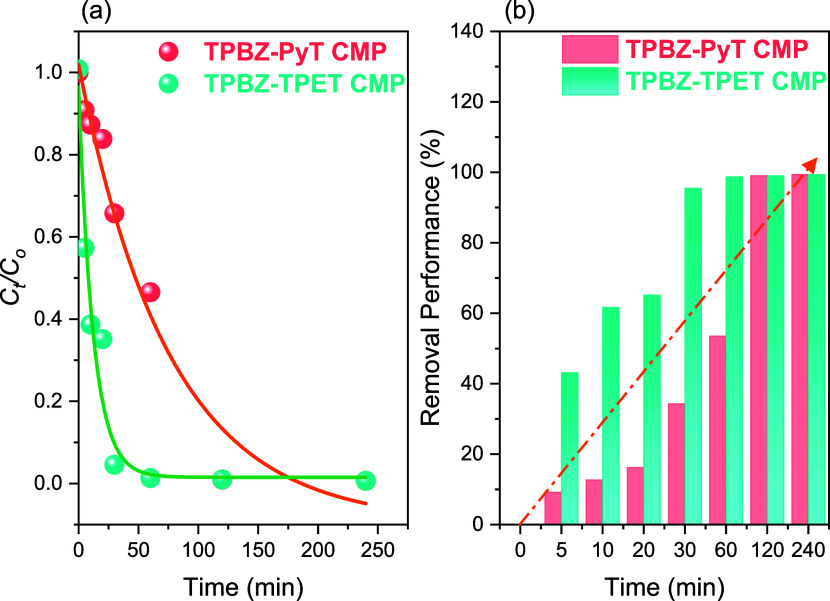
Change of *C_t_*/*C*_0_ as a function of adsorption time (a) and (b) adsorption removal performances of TPBZ-PyT CMP and TPBZ-TPET CMP.

TPBZ-TPET CMP, with its higher surface area and porous structure, offers a greater number of active sites for RhB adsorption. This enhanced surface area facilitates strong interactions, such as π–π stacking and hydrogen bonding, which are critical for efficient dye adsorption. Furthermore, the flexible TPE moiety in TPBZ-TPET CMP likely improves its ability to accommodate RhB molecules, thereby increasing its adsorption capacity. Additionally, adsorption kinetics play a crucial role in determining dye removal efficiency. The rapid initial uptake of RhB observed with TPBZ-TPET CMP suggests highly favorable interactions at its surface, enabling quick engagement with RhB molecules. In contrast, the slower adsorption rate of TPBZ-PyT CMP may be attributed to its more rigid structure, which limits the accessibility of adsorption sites and reduces its interaction efficiency with RhB. These findings underline the importance of material selection in the design of effective adsorbents for dye removal from aqueous solutions. The superior performance of the TPBZ-TPET CMP not only emphasizes its potential for practical applications in wastewater treatment but also suggests that structural characteristics, such as surface area and flexibility, are crucial determinants of adsorption efficiency. The adsorption kinetics of RhB onto TPBZ-PyT and TPBZ-TPET CMPs were evaluated using pseudo-first-order, pseudo-second-order, and intraparticle diffusion models to determine the potential rate-controlling steps in the adsorption process. The experimental data were fitted to the linear equations corresponding to these models, as outlined in eqs 3–5.

3 $$\log\left(Q_{e}-Q_{t}\right)=\log\left(Q_{e}\right)-\frac{k_{1}t}{2.303}$$

4 $$\frac{t}{Q_{t}}=({k_{2}Q_{e}^{2})}^{-1}+t/Q_{e}$$

5 $$Q_{t}=K_{i}t^{1/2}+C$$

The pseudo-first-order model, also known as the Lagergren model, assumes that the adsorption rate is directly proportional to the number of unoccupied adsorption sites. In contrast, the pseudo-second-order model considers both the adsorption capacity of the adsorbent and the amount of adsorbate adsorbed at equilibrium. The correlation coefficients (*R* ^2^) obtained from linear regression analysis provide insights into the suitability of these models for describing the adsorption kinetics. For TPBZ-TPET CMP, the pseudo-second-order model demonstrated a higher *R* ^2^ value of 0.994 (Table S2), indicating that this model more accurately represents the adsorption kinetics. Conversely, for TPBZ-PyT CMP, the pseudo-first-order model exhibited a higher *R* ^2^ value of 0.978 (Table S2), suggesting that the adsorption process for this material follows a pseudo-first-order mechanism. The equilibrium adsorption capacities (*Q*_e_) calculated using the pseudo-first-order model were 41.946 mg g^–1^ for TPBZ-PyT CMP and 48.367 mg g^–1^ for TPBZ-TPET CMP [Figure 8a and Table S2]. In contrast, the pseudo-second-order model yielded higher Q_e_ values of 103.51 mg g^–1^ for TPBZ-PyT CMP and 55.55 mg g^–1^ for TPBZ-TPET CMP [Figure 8b and Table S2]. The observed discrepancy between the experimental and calculated *Q*_e_ values suggests that the pseudo-second-order model may not fully capture the complexity of the adsorption process (Table S2). The rate constants (*K*_1_ and *K*_2_) provide valuable insights into the adsorption kinetics. TPBZ-TPET CMP demonstrated a higher pseudo-first-order rate constant (*K*_1_ = 0.0855 min^–1^) compared to TPBZ-PyT CMP (*K*_1_ = 0.0128 min^–1^), indicating a faster initial adsorption rate [Figure 8a and Table S2]. Similarly, in the pseudo-second-order model, TPBZ-TPET CMP exhibited a higher rate constant (*K*_2_ = 2.54 × 10^–3^ g mg^–1^ min^–1^) compared to TPBZ-PyT CMP (*K*_2_ = 4.95 × 10^–5^ g mg^–1^ min^–1^), further confirming the superior adsorption kinetics of TPBZ-TPET CMP [Figure 8b and Table S2]. The elevated correlation coefficient of the pseudo-second-order model for TPBZ-TPET CMP suggests that the adsorption process is predominantly governed by chemisorption, involving valence interactions through electron sharing or exchange between the adsorbent and adsorbate.^55^ Conversely, the adsorption behavior of TPBZ-PyT CMP corresponds to a pseudo-first-order model, indicating that the adsorption rate is primarily influenced by the number of available adsorption sites. The kinetic data underscores the differences in adsorption mechanisms between TPBZ-PyT and TPBZ-TPET CMPs. TPBZ-TPET CMP exhibits faster adsorption kinetics and higher adsorption capacity, likely attributed to its larger surface area and favorable interactions with RhB molecules. While the pseudo-second-order model accurately describes the adsorption process for TPBZ-TPET CMP, the adsorption kinetics of TPBZ-PyT CMP align with the pseudo-first-order model. The intraparticle diffusion model (eq 5) offers additional insights into the adsorption behavior of RhB on TPBZ-PyT and TPBZ-TPET CMPs.

**Figure 8:**
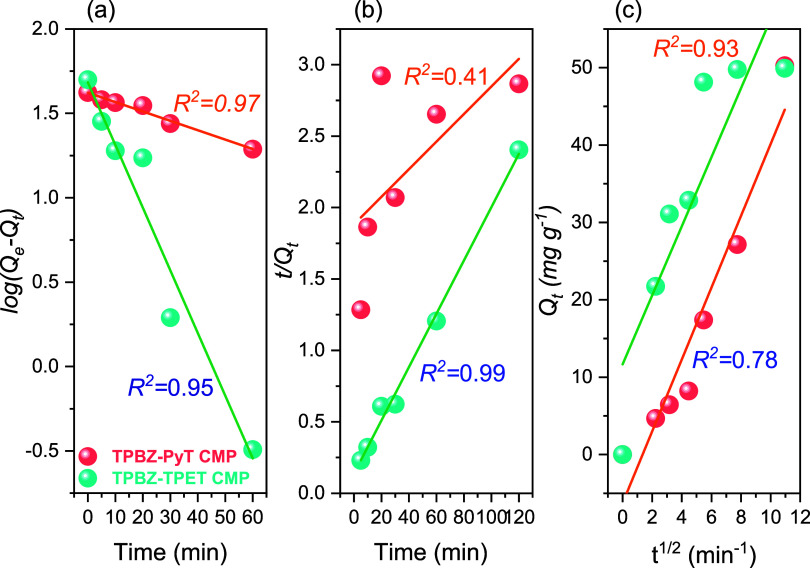
(a) Pseudo-first-order, (b) pseudo-second-order, and (c) intraparticle diffusion assumptions modeling of the removal of RhB species utilizing the TPBZ-PyT CMP and TPBZ-TPET CMP.

This model explains adsorption in porous materials, where the uptake of the adsorbate is proportional to the square root of time if intraparticle diffusion acts as the rate-controlling step. The linear regression plots of Q_t_ against *t* ^1/2^ for the experimental data [Figure 8c] reveal several key observations. During the initial stages, the plots are linear for both CMPs, indicating rapid adsorption of RhB molecules onto the external surfaces. This linear phase suggests that surface adsorption is the dominant mechanism during the early stages of the process. However, the plots for both TPBZ-PyT and TPBZ-TPET CMPs deviate from passing through the origin, suggesting that intraparticle diffusion is not the only factor governing the rate of the adsorption process. This deviation suggests that other mechanisms, such as external surface adsorption, also contribute to the overall adsorption process. Following the initial rapid adsorption, the plots transition to a nonlinear phase, reflecting the diffusion of RhB molecules into the microporous framework of the CMPs. During this stage, the RhB molecules penetrate the internal pore structure. As the micropore volume becomes saturated, the adsorption process stabilizes, maintaining a fairly constant rate. The intercepts of the linear plots offer insights into the thickness of the boundary layer. For TPBZ-PyT and TPBZ-TPET CMPs [Figure 8c], the intercepts are 4.640 and 4.459 mg g^–1^, respectively. These nonzero intercepts highlight the significance of the boundary layer effect, emphasizing the critical role of surface adsorption in the overall adsorption process. The intraparticle diffusion rate parameter (*K_i_*) is determined from the slope of the linear portion of the plots. The K_i_ values (Table S2) provide valuable information about the rate of adsorption within the porous structures of the CMPs. A higher *K_i_* value indicates faster intraparticle diffusion, leading to an enhanced adsorption rate within the CMPs. The adsorption kinetics of RhB onto the TPBZ-PyT and TPBZ-TPET CMPs demonstrate distinct differences in the rate of reduction, as evidenced by the equivalent concentration percentages (*C_t_*/*C*_0_) over time intervals and the pseudo-first-order kinetic model. To further investigate the adsorption kinetics, the pseudo-first-order reduction kinetics model was applied, as expressed in eq 6. The linear relationship between ln­(*C_t_*/*C*_0_) and time (*t*) is illustrated in Figure S14. The kinetic rates of reduction were determined to be 0.012 min^–1^ for TPBZ-PyT CMP and 0.077 min^–1^ for TPBZ-TPET CMP.

6 $$\mathrm{Pseudo}‐\mathrm{first}‐\mathrm{order}\;\mathrm{reduction}\;\mathrm{model}:\ln\left(C_{t}/C_{o}\right)=-\mathrm{kt}$$

The significantly higher rate of reduction observed in TPBZ-TPET CMP can be attributed to its superior structural characteristics and adsorption properties. TPBZ-TPET CMP likely features a larger surface area and a more favorable pore structure, offering an increased number of active sites for RhB adsorption.

Additionally, the incorporation of TPE into the TPBZ-TPET CMP enhances its flexibility, enabling it to better accommodate RhB molecules and thus achieve faster adsorption kinetics. Moreover, the presence of multiple phenyl groups in the TPE moiety promotes stronger π–π stacking interactions and hydrogen bonding with RhB molecules. These enhanced interactions further accelerate the adsorption process, contributing to the higher rate of reduction observed for TPBZ-TPET CMP compared to TPBZ-PyT CMP. The adsorption behavior of RhB dye onto the TPBZ CMPs was further analyzed using the Langmuir isothermal model (eq 1) to fit the adsorption data. Based on the relationship between *C*_e_/*Q*_e_ and *C*_e_ [Figure 9a], the linear fit exhibited high correlation coefficients (*R*_L_^2^) of 0.993 and 0.986 for TPBZ-PyT and TPBZ-TPET CMPs, respectively. According to the Langmuir isotherm models [Figure 9a,b, and Table S3], the maximum adsorption capacities (*Q*_m_) for TPBZ-PyT and TPBZ-TPET CMPs were determined to be 421 and 704.22 mg g^–1^, respectively. Additionally, the Freundlich isothermal model (eq 2) was employed to provide further insights into the interactions between RhB molecules and the TPBZ CMPs [Figure 9c]. A summary of the results, including the key parameters, is presented in Table S3, offering a clearer understanding of the chemical interactions involved in the adsorption process. The correlation coefficients (*R*_F_^2^) for the adsorption of RhB on TPBZ-PyT and TPBZ-TPE CMPs were 0.972 and 0.986, respectively, according to the Freundlich isothermal models (Table S3). However, the higher correlation coefficients observed for the Langmuir isotherm models compared to the Freundlich models indicate that RhB adsorption on TPBZ CMPs predominantly occurs as monolayer coverage. This finding validates the homogeneous surface characteristics of the TPBZ CMPs. The results also highlight that TPBZ-TPET CMP exhibits superior adsorption capacity compared to TPBZ-PyT CMP. This enhanced performance can be attributed to stronger π–π stacking interactions, larger surface area, and higher permeability of TPBZ-TPET CMP. Repeatability adsorption trials were conducted for TPBZ-PyT and TPBZ-TPET CMPs under identical initial conditions to validate their practical applicability. Remarkably, no significant performance losses were observed after five consecutive adsorption cycles (Figure S15). This demonstrates the reusability and stability of the TPBZ CMPs. Additionally, the excellent chemical stability of the CMPs was confirmed through a close match of their FTIR spectra before and after the adsorption tests. The FTIR spectra of the TPBZ CMPs (Figure S16) offer valuable evidence of π–π stacking interactions between the TPBZ CMPs and RhB molecules, aligning with similar observations reported for various RhB adsorbents.^45^ As illustrated in Figure S16, only slight shifts in wavenumbers were detected, with no significant alterations in the FTIR peaks, reinforcing the hypothesis that the interaction between the dye molecules and the adsorbents is predominantly physical (π–π stacking). Additionally, a peak at 1553 cm^–1^, indicative of aryl C=C bonds, is clearly visible in the FTIR spectrum of the RhB dyes. After the adsorption process, no apparent changes in the CMPs’ FTIR bands were detected, aside from slight peak shifts in the C=C bonds. To further evaluate the performance of our TPBZ CMPs for removing RhB dye from water, we compared the *Q*_m_ values of TPBZ-TPE and TPBZ-PyT CMPs with those of other advanced polymeric materials (Table S4).

**Figure 9:**
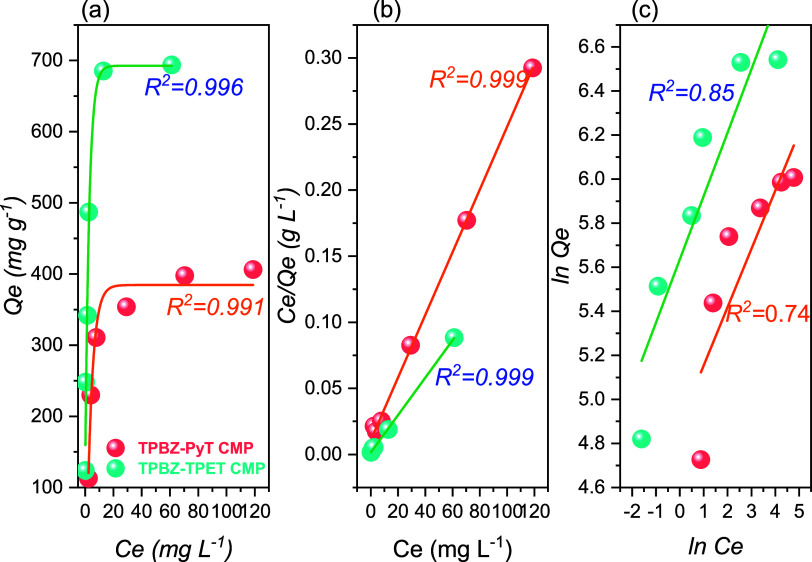
(a) Adsorption capacities, (b) Langmuir, and (c) the Freundlich isotherm assumptions utilizing TPBZ-PyT CMP and TPBZ-TPET CMP depending on different starting percentages of RhB dyestuff.

### Thermodynamic Studies of TPBZ CMPs Adsorbents

The adsorption of RhB dye onto TPBZ CMPs is strongly influenced by temperature, a key thermodynamic factor. To investigate this, RhB dye adsorption experiments were conducted using 5 mg of TPBZ CMPs in 10 mL of a 55 mg L^–1^ RhB solution at four different temperatures: 303, 323, 333, and 343 K (Figure S17). A noticeable decrease in RhB adsorption capacity was observed for the TPBZ CMPs at higher temperatures. For TPBZ-PyT CMP, the adsorption capacities were 110.26, 106.28, 104.21, and 73.03 mg g^–1^ at 303, 323, 333, and 343 K, respectively. Similarly, TPBZ-TPET CMP exhibited adsorption capacities of 106.71, 99.92, and 93.66 mg g^–1^ as the temperature increased across the same range. The exothermic nature of RhB adsorption onto TPBZ CMPs is evident from the reduced adsorption efficiency at higher temperatures. This behavior can be attributed to the weakened interactions between RhB molecules and the TPBZ CMPs as the temperature increases. Key thermodynamic parameters, including Gibbs free energy (ΔG°), adsorption enthalpies (Δ*H*°), and entropy change (Δ*S*°), can be quantitatively determined using the experimental results and eqs 7–5. In these equations, K_d_ represents the thermodynamic equilibrium constant.

7 $$\Delta G^{{}^{\circ}}=-\mathrm{RT}\;\ln K_{d}$$

8 $$\ln K_{d}=\frac{\Delta S^{{}^{\circ}}}{R}-\frac{\Delta H^{{}^{\circ}}}{\mathrm{RT}}$$

9 $$K_{d}=\frac{q_{e}}{C_{e}}$$

The spontaneous binding of RhB onto TPBZ CMPs is confirmed by the negative values of Δ*G*°, as illustrated in Figure S17. Additionally, Δ*H*° and Δ*S*° values were determined using the slope and intercept of the linear plot of ln *K*_d_ against 1/T. These results provide insight into the mechanism of RhB physisorption onto TPBZ-PyT and TPBZ-TPET CMPs. The Δ*H*° values for TPBZ-TPET and TPBZ-PyT CMPs adsorbents were found to be −83.44 and −79.02 kJ moL^–1^, respectively, confirming the exothermic nature of RhB adsorption onto TPBZ CMPs. The Δ*S*° values for TPBZ-PyT and TPBZ-TPET CMPs adsorbents were −0.211 and −0.227 J mol^–1^ K^–1^, respectively, which suggests a reduction in the disorder level across the interfaces between CMPs and liquids without causing a major alteration to the interior frameworks of TPBZ CMPs. These results imply a robust π–π stacking between RhB dyestuff and TPBZ CMPs. The *R* ^2^ values for the Van’t Hoff plots of both TPBZ-PyT CMP and TPBZ-TPET CMP CMPs are 0.915 and 0.992, respectively [Figure S17]. The *R* ^2^ value for TPBZ-TPET CMP demonstrates an excellent linear correlation, indicating highly reliable thermodynamic parameter extraction. For TPBZ-PyT CMP, the *R* ^2^ value of 0.915, while somewhat lower, still indicates a reasonably strong correlation, but we acknowledge that the fit is less robust than for TPBZ-TPET CMP. This may reflect subtle differences in adsorption behavior or heterogeneity in the adsorption sites of TPBZ-PyT CMP at different temperatures.

### DFT Calculations

Density functional theory (DFT) was employed to examine the electrical characteristics, chemical reactivity, and adsorption properties of the synthesized TPBZ CMPs. This investigation employed the DMol3 module,^56^ utilizing the Generalized Gradient Approximation (GGA).^57^ Geometry optimization calculations were performed using the DNP basis set and the Perdew–Wang exchange and correlation functional (PW91).^58,59^ To comprehend the electronic structures of the molecules, it is necessary to analyze their electronic structure as well as their frontier molecular orbitals. Table S5 provides the calculated values for the HOMO (highest occupied molecular orbital) and LUMO (lowest unoccupied molecular orbital) energies. Both TPBZ CMP exhibit almost identical HOMO energies (−4.68 eV for TPBZ-PyT CMP and −4.69 eV for TPBZ-TPET CMP), reflecting a similar potential for electron donation. In contrast, the LUMO energy of TPBZ-PyT CMP is lower (−2.15 eV) compared to TPBZ-TPET CMP (−1.87 eV), which results in a reduced band gap (Δ*E*) of 2.53 eV for TPBZ-PyT CMP, as opposed to 2.82 eV for TPBZ-TPET CMP. Figure 10a shows the optimized structures of polymers and dye molecules. As can be seen in Figure 10b, the donor units (TPBZ) of TPBZ-TPET and TPBZ-PyT CMPs predominantly determine the HOMO levels, while the acceptor units (Py or TPE) influence the LUMO levels. The twisted TPE unit in TPBZ-TPET CMP is likely to exert a stronger influence on orbital overlap and delocalization than the planar pyrene structure in TPBZ-PyT CMP. For RhB dye, the electron density in the HOMO is distributed over the conjugated π-system, primarily the xanthene moiety and phenyl ring, while the LUMO is localized mainly on the phenyl ring. Table S5 displays the electrical properties of the global reactivity parameters. The HOMO and LUMO energy eigenvalues were used to calculate the reactivity indices. The susceptibility of polymers to electron gains or loss is shown by this parameter. The electron affinity (A), representing the tendency to accept electrons, is marginally greater for TPBZ-PyT CMP (2.15 eV) compared to TPBZ-TPET CMP (1.87 eV). TPBZ CMPs have almost the same ionization potential. In comparison to TPBZ-TPET CMP, which has a higher chemical hardness (η) of 1.41 eV, TPBZ-PyT CMP has a lower value of 1.27 eV. This suggests that TPBZ-PyT CMP could exhibit greater reactivity in both electron donation and acceptance processes. Unlike TPBZ-TPET CMP, which is somewhat hard, TPBZ-PyT CMP is quite soft. The electrophilicity index (ω) and electronegativity (χ) of TPBZ-PyT CMP show that it can accept electrons more strongly than TPBZ-TPET CMP. Finally, the chemical potential (μ) represents the inclination to either loss or gain electrons. A negative chemical potential signifies that TPBZ CMPs tend to attract electrons. The electrostatic potential (ESP) maps of TPBZ-PyT CMP and TPBZ-TPET CMP reveal that the two CMPs share a similar electronic structure, with slight differences caused by the differing geometries of the acceptor units [Figure S18]. The abundance of electrons in red regions (negative ESP) is a result of the presence of electronegative N and O atoms. Areas with a low electron density, known as blue regions (positive ESP), may encircle aromatic rings and H atoms. The level of potential increases in the order red < orange < yellow < green < blue. The ESP surfaces of TPBZ-PyT CMP and TPBZ-TPET CMP [Figure S18a and S18b] show a highly conjugated and extended π-system, suggesting that electron density is delocalized throughout the entire molecular structure. Their optoelectronic properties can be enhanced by significant delocalization. The presence of aromatic groups influences the overall electronic distribution, as seen by the increased electron density surrounding the Py moieties and TPE units in TPBZ-PyT CMP [Figure S18a] and TPBZ-TPET CMP [Figure S18b], respectively. Figure S18c shows that the RhB dye has a negative potential near the nitrogen atoms and carboxyl groups, as well as a greater electron density surrounding the xanthene core. The Fukui index study reveals the reactivity of RhB dye. Atoms such as C(6), C(11), C(12), C(18), C(19), and C(32) have high Fukui indices for electrophilic attack (f – ), as shown in Table S6, suggesting a high exposure to electrophilic interactions. Nevertheless, the high nucleophilic attack (f + ) indices of C(6), C(11), C(12), C(31), and C(32) atoms are very indicative of nucleophilic reactions. Molecular dynamics simulations were performed at room temperature to investigate the interactions between the adsorbent and adsorbate. As shown in Table S7, Monte Carlo (MC) simulation results indicate that the interaction energy is the highest between RhB and TPBZ-PyT CMP and TPBZ-TPET CMP. The negative interaction energies observed in all systems confirm the presence of strong attractive interactions.

**Figure 10:**
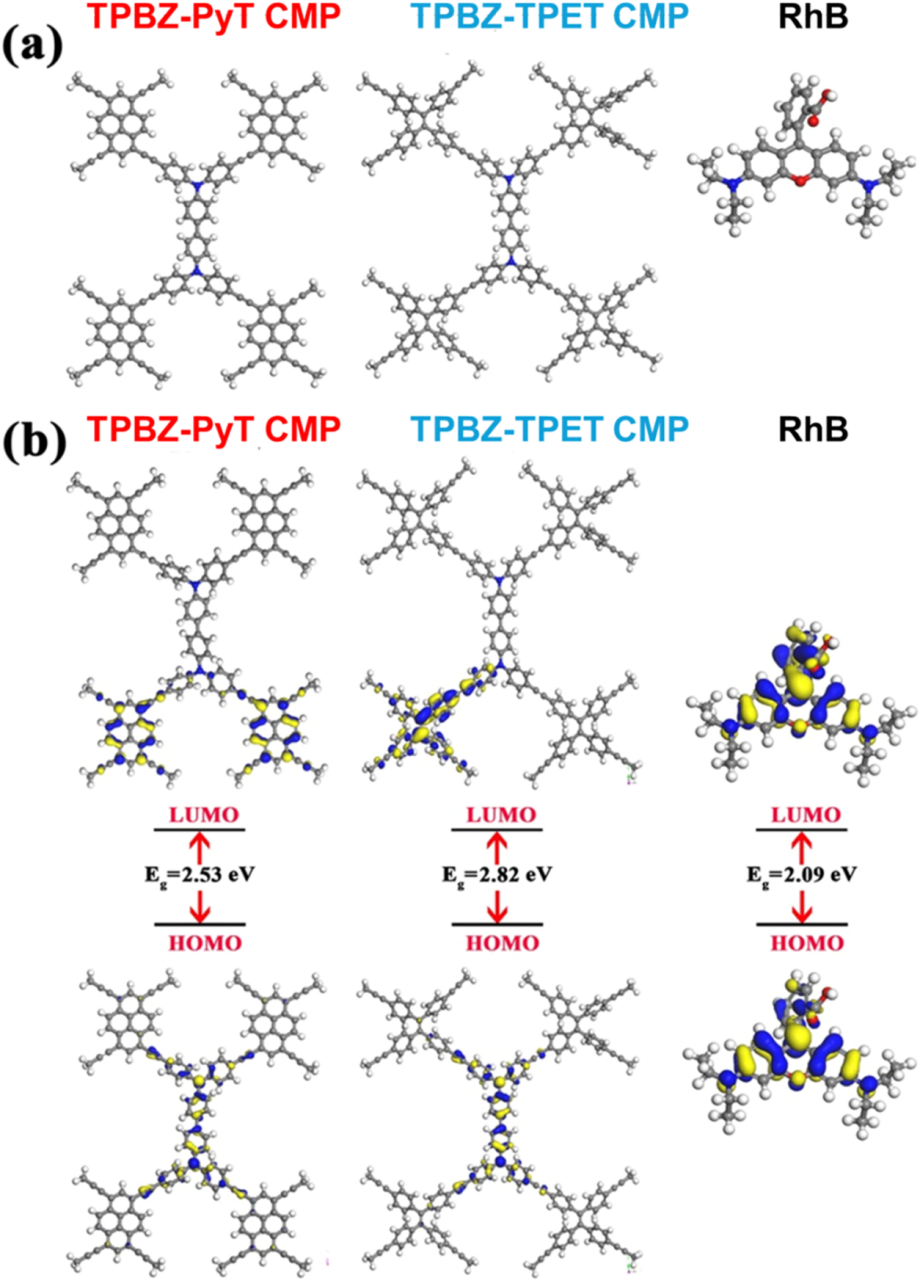
(a) Optimized structure, (b) Frontier molecular orbitals, and HOMO–LUMO bandgap energies of the TPBZ-PyT CMP, TPBZ-TPET CMP, and RhB.

Figure S19 presents the adsorption energies of RhB dye on the studied molecules, revealing values of −78.83 kcal/mol for TPBZ-PyT CMP and −79.70 kcal/mol for TPBZ-TPET CMP. These results suggest that π–π stacking and electrostatic interactions play a crucial role in RhB adsorption, with TPBZ-TPET CMP exhibiting slightly stronger binding than TPBZ-PyT CMP. The robust adsorption of RhB observed in the simulations aligns well with experimental findings, further validating the adsorption mechanism. In conclusion, the investigation of the electrical properties, chemical reactivity, and adsorption behavior of TPBZ-PyT CMP and TPBZ-TPET CMP using DFT and MC simulations has provided valuable insights. The TPBZ-TPET CMP exhibited superior RhB dye adsorption properties, attributed to its larger BET surface area and stronger adsorption energy. The comparative analysis of these CMPs revealed that TPBZ-TPET CMP interacts more strongly with RhB dye than TPBZ-PyT CMP.

## Conclusions

This study examined the adsorption performance of two novel CMPs, TPBZ-PyT CMP and TPBZ-TPET CMP, for the removal of RhB dye from aqueous solutions. TPBZ-TPET CMP featured the flexible TPE subunit, while TPBZ-PyT CMP incorporated the more rigid Py moiety. Characterization confirmed that TPBZ-TPET CMP possessed a higher surface area, contributing to its superior adsorption capacity. Batch adsorption experiments demonstrated that TPBZ-TPET CMP achieved a remarkable 98.72% RhB removal within 60 min, significantly outperforming TPBZ-PyT CMP, which removed 53.49% of RhB under similar conditions. Kinetic studies indicated that TPBZ-TPET CMP followed a pseudo-second-order mechanism, suggesting chemisorption as the primary rate-controlling step. Conversely, TPBZ-PyT CMP adhered to a pseudo-first-order mechanism. These findings underscore the critical role of subunit flexibility in enhancing the adsorption efficiency of CMPs. This research contributes to the development of advanced materials for efficient water purification, offering a promising solution to address dye contamination in aquatic environments.

## Supplementary Material



## Notes

Characterization, synthesis; FTIR and NMR spectroscopy of PyT and TPET; XPS and XRD analyses of TPBZ-PyT and TPBZ-TPET CMPs; SEM-EDS images of TPBZ-PyT CMP; the effect of the contact time on the adsorption properties of TPBZ-PyT and TPBZ-TPET CMPs; plots of ln­(*C_t_*/*C*_0_) against the reaction time for reducing RhB dyestuff by TPBZ-PyT and TPBZ-TPET CMPs; recycling profile of TPBZ-PyT and TPBZ-TPET CMPs toward RhB dyestuff adsorption; FTIR profiles of pristine and recycled TPBZ-PyT and TPBZ-TPET CMPs; Van’t Hoff plots for RhB adsorption onto TPBZ-PyT and TPBZ-TPET CMP; electrostatic potential (ESP) distributions of TPBZ-PyT CMP, TPBZ-TPET CMP, and RhB dye; the most stable configuration of RhB dye adsorption over TPBZ-PyT CMP and RhB dye adsorption over TPBZ-TPET CMP (PDF)
