# Supplementary material for: Unlocking the Potential of N,N,N′,N′‑Tetraphenylbenzidine Based on Conjugated Microporous Polymers for Rhodamine B Adsorption: A Synergistic Experimental and Density Functional Theory Perspective
Source: ACS Polym Au. 2025 Jun 10;5(4):379–93. doi: 10.1021/acspolymersau.5c00025 (PMC12355617; doi:10.1021/acspolymersau.5c00025)
Supplement: Supplementary file 1 [file lg5c00025_si_001.pdf]

Supporting Information

Unlocking the Potential of N,N,N′,N′-Tetraphenylbenzidine Based on Conjugated Microporous Polymers for Rhodamine B Adsorption: A Synergistic Experimental and Density Functional Theory Perspective

Mohammed G. Kotp[a1], Mohamed Gamal Mohamed[a,b1]*, Pei-Tzu Wang[a], Ahmed E. Hassan[c,d], Ahmed M. Elewa[e], and Shiao-Wei Kuo[a,f]*

[a]Department of Materials and Optoelectronic Science, Center for Functional Polymers and Supramolecular Materials, National Sun Yat-Sen University, Kaohsiung 804, Taiwan.

[b]Chemistry Department, Faculty of Science, Assiut University, Assiut 71515, Egypt.

[c]Interdisciplinary Research Center for Hydrogen Technologies and Carbon Management (IR-CHTCM), King Fahd University of Petroleum & Minerals, Dhahran 31261, Saudi Arabia.

[d]Department of Chemistry, Faculty of Science, Al-Azhar University, Nasr City 11884, Cairo, Egypt.

[e]Department of Chemical Engineering, National Tsing Hua University, Hsinchu 300044, Taiwan.

[f]Department of Medicinal and Applied Chemistry, Kaohsiung Medical University, Kaohsiung 807, Taiwan.

Corresponding authors:

E-mail: mgamal.eldin12@gmail.com (M. G. Mohamed) and kuosw@faculty.nsysu.edu.tw (S. W. Kuo).

[1]These authors equally contributed to this work.

Characterization

FTIR spectra were collected on a Bruker Tensor 27 FTIR spectrophotometer with a resolution of 4 cm^{-1} by using the KBr disk method. ^{13}C nuclear magnetic resonance (NMR) spectra were examined by using an INOVA 500 instrument with DMSO-d_6 and $CHCl_3$-d as the solvent and TMS as the external standard. Chemical shifts are reported in parts per million (ppm). The thermal stabilities of the samples were performed by using a TG Q-50 thermogravimetric analyzer under a N_2 atmosphere; the sample (ca. 5 mg) was put in a Pt cell with a heating rate of 20 °C min^{-1} from 100 to 800 °C under a N_2 flow rate of 60 mL min^{-1}. Solid-state ^{13}C NMR was measured by JEOL JNM-LA300 spectrometer and standard CPMAS probe at 75.577 MHz. The morphologies of the polymer network samples were examined by Field emission scanning electron microscopy (FE-SEM; JEOL JSM7610F) and transmission electron microscope (TEM) using a JEOL-2100 instrument at an accelerating voltage of 200 kV. Surface area and porosity measurements of samples weighing approximately 40-60 mg were conducted using the BEL MasterTM/BEL simTM (version 3.0.0) apparatus. Nitrogen (N_2) adsorption and desorption isotherms were generated by gradually exposing the samples to ultrahigh-purity N_2 gas, reaching pressures of up to about 1 atmosphere, while maintaining a temperature of 77 K in a liquid nitrogen bath. Before these measurements, the samples underwent a degassing process at 150 °C for 8 h. The instrument's software was utilized to calculate surface parameters using the BET adsorption models. Furthermore, the pore size of the prepared samples was determined using nonlocal density functional theory (NLDFT).

Synthesis of 1,3,6,8-tetraethynylpyrene [PyT]

A total of 1.00 g of pristine py (equivalent to 5 mmol) was dissolved in 10 mL of nitrobenzene. Subsequently, a solution containing bromine (1.15 mL, 22 mmol) in 10 mL of nitrobenzene was gradually added to the py suspension. The mixture was then heated at 120 °C for 4 h. After cooling to ambient temperature, the resulting product was filtered and washed with ethanol. The solid was then placed in an oven at 60 °C for 24 h, yielding a yellowish-white solid (designated as Py-4Br) weighing 2.20 g, representing a yield of 90%. The FT-IR analysis (KBr) revealed absorption peaks at 3053 cm^{-1} (indicative of aromatic C–H stretching) and 682 cm^{-1} (associated with C–Br stretching). Subsequently, under a N_2 environment, a mixture was prepared by combining 220 mg (0.12 mmol) of $Pd(PPh_3)_4$, 244 mg (0.92 mmol) of PPh_3, 118 mg (0.62 mmol) of CuI, and 2 g (2.38 mmol) of the synthesized Py-4Br in 28 mL of toluene along with 28 mL of triethylamine. This mixture was heated to 50 °C, after which TMSA (2.34 g, 23.8 mmol) was added dropwise. The reaction was maintained at 90 °C for two nights. Once the reaction was complete, the mixture was cooled to ambient temperature, and the solid was separated from the solvent by filtration through Celite affording (Py-TMS). FT-IR analysis indicated peaks at 3053 cm^{-1} (aromatic C–H stretching), 2908 cm^{-1} (aliphatic C–H stretching), 2100 cm^{-1} (C ≡ C stretching), and 1618 cm^{-1} (C=C stretching). The ^1H NMR spectrum (500 MHz, δ, ppm, $CDCl_3$) showed signals at 0.413 (s, 36H, CH_3), 8.3 (s, 2H), and 8.57 (s, 4H). The ^{13}C NMR spectrum (125 MHz, δ, ppm, $CDCl_3$) revealed chemical shifts at 135.70, 132.40, 127.80, 119.20, 103.50, and 101.60. Ultimately, a solution of the Py-TMSA (2.00 g, 3.41 mmol) and K_2CO_3 (5.70 g, 42 mmol) was prepared in 50 mL of anhydrous methanol. The reaction was allowed to proceed at room temperature for 48 h. After this period, the methanol was evaporated under reduced pressure, yielding PyT as a sun-orange powder (1.88 g, 94.30% yield). FT-IR analysis (KBr, cm^{-1}) showed characteristic peaks at 3279 cm^{-1} (≡C-H), 3065 cm^{-1} (aromatic C–H stretching), 2186 cm^{-1} (C ≡ C stretching), and 1618 cm^{-1} (C=C stretching). The ^1H NMR spectrum (500 MHz, δ, ppm, $CDCl_3$) displayed signals at 8.68 (s, 4H), 8.38 (s, 2H), and 3.67 (s, 4H). Additionally, the ^{13}C NMR

spectrum (125 MHz, δ, ppm, $CDCl_3$) revealed chemical shifts at 133.80, 130.80, 129.10, 127.80, 84.50, and 59.70 ppm.

Synthesis of 1,1,2,2-tetrakis(4-ethynylphenyl)ethene [TPET]

benzophenone (3.00 g, 16.4 mmol) and zinc (4.31 g, 65.9 mmol) were combined in 80 mL of THF and stirred in an ice bath for 10 minutes. $TiCl_4$ (3.60 mL, 33.0 mmol) was then added wisely over 30 minutes. The mixture was subsequently warmed to 80 °C and maintained under reflux. After the reaction, 5% aqueous K_2CO_3 was introduced. The organic solvent was removed under reduced pressure, and the remaining solution was extracted three times with ethyl acetate (EtOAc) and water. The solution was dried over magnesium sulfate to eliminate any Waterous molecules, and after evaporating the EtOAc, the residue was washed with ethanol, resulting in a white solid known as TPE (2.66 g, 97% yield). The melting point of 228–229 °C (DSC). FT-IR analysis (KBr, cm^{-1}) showed peaks at 3047 (aromatic C–H stretching) and 1602 (C=C stretching). The ^{1}HNMR spectrum (500 MHz, $CDCl_3$) displayed signals at 7.26 (d, 8H) and 6.84 (d, 8H). The ^{13}CNMR spectrum (125 MHz, $CDCl_3$) revealed chemical shifts at 140.70, 141.00, 131.30, 127.70, and 126.40 ppm. In a separate procedure, the designed TPE (3.32 g, 10.0 mmol) was mixed with 25 mL of acetic acid and 50 mL of DCM in a 100 mL flask and kept in an ice bath. Bromine (10.00 mL, 200.0 mmol) was charged, and the mixture was stirred at room temperature for 48 h. The organic solvents were then extracted with DCM and water. The organic layer was dried over $MgSO_4$, and DCM was removed under vacuum. The resulting substance was recrystallized from methanol to obtain a crystalline white powder. To purify the product, the white powder was dissolved in 350 mL of DCM, filtered to remove impurities, and then 250 mL of methanol was added. After evaporating the DCM on a hotplate at 70 °C, the white solid collected was identified as TPE-4Br (6.15 g, 95% yield). The melting point was recorded at 261–262 °C (DSC). FT-IR analysis (KBr, cm^{-1}) indicated peaks at 3051 (aromatic C–H stretching) and 1572 (C=C stretching). The ^{1}H NMR spectrum (500 MHz, $CDCl_3$) showed signals at 7.25 (d,

8H) and 6.84 (d, 8H), while the ^{13}C NMR spectrum (125 MHz, $CDCl_3$) displayed shifts at 142.30, 139.70, 133.70, 131.90, and 121.80 ppm. In a nitrogen atmosphere, TPE-4Br (2.00 g, 3.09 mmol), TMSA (2.43 g, 24.70 mmol), $Pd(PPh_3)_4$ (0.17 g, 0.247 mmol), CuI (0.094 g, 0.494 mmol), and PPh_3 (0.194 g, 0.741 mmol) were combined with 25 mL of THF and 25 mL of Et_3N in a 500 mL flask. The mixture was filtered and washed with THF to obtain the resulting solution. The organic solvent was then removed using a rotary evaporator, followed by extraction with DCM and deionized water, and dehydration with $MgSO_4$. The resulting white solid, after separation and rinsing three times with water, was identified as TPE-TMSA. FTIR analysis (KBr, cm^{-1}) showed characteristic peaks at 3060 (aromatic C–H stretching), 2920 (aliphatic C–H stretching), 2155 (C≡C stretching), and 1618 (C=C stretching). The ^{1}H NMR spectrum (500 MHz, $CDCl_3$, δ, ppm) revealed signals at 7.24 (d, J = 8.4 Hz, 8H), 6.88 (d, J = 8.4 Hz, 8H), and 0.22 (s, 36H, CH_3). The ^{13}C NMR spectrum (125 MHz, $CDCl_3$, δ, ppm) showed shifts at 144, 141, 132.7, 132, 122.3, 105.6, 95.8, and 0.07. Subsequently, TPE-TMSA (1.5 g) and an excess of K_2CO_3 (3.0 g) were stirred in 70 mL of methanol in a round-bottom flask, which was sealed with a rubber septum and allowed to react at room temperature over two nights. The precipitate was washed with water and methanol, then dried at 70 °C for 24 h to yield TPET as a misty yellow solid.

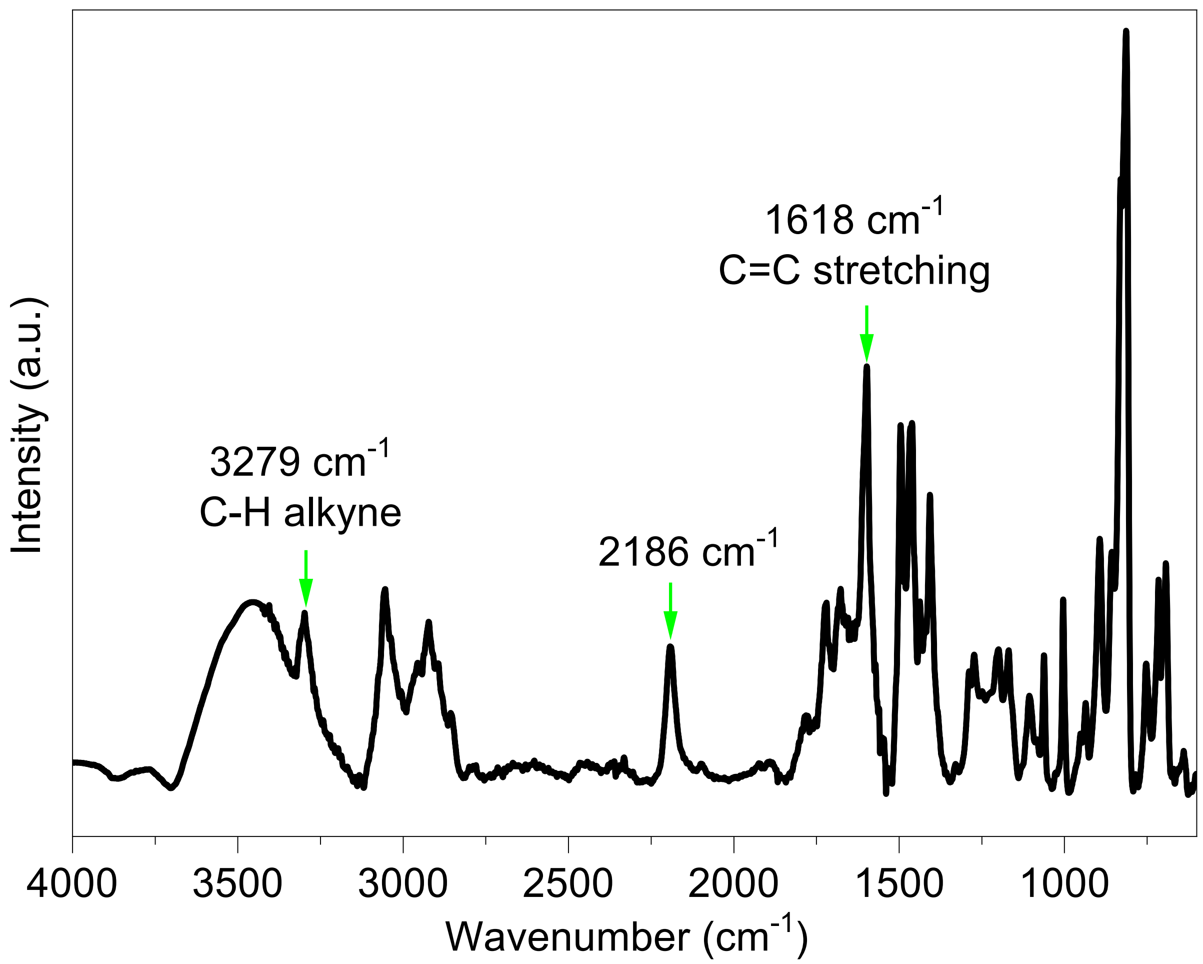

Figure S1. FTIR profile of PyT.

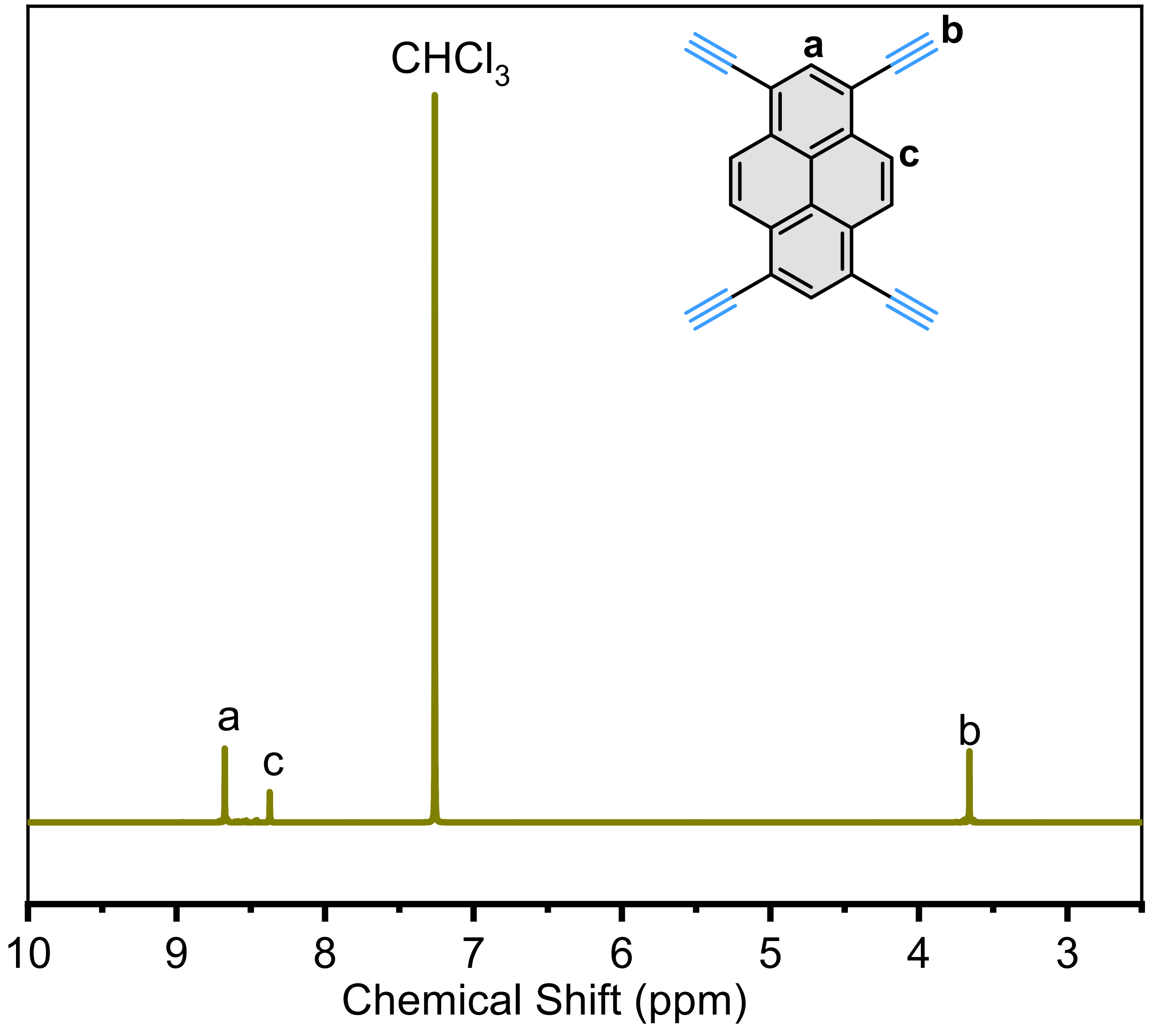

Figure S2. ^{1}H-NMR spectrum of PyT in $CDCl_3$.

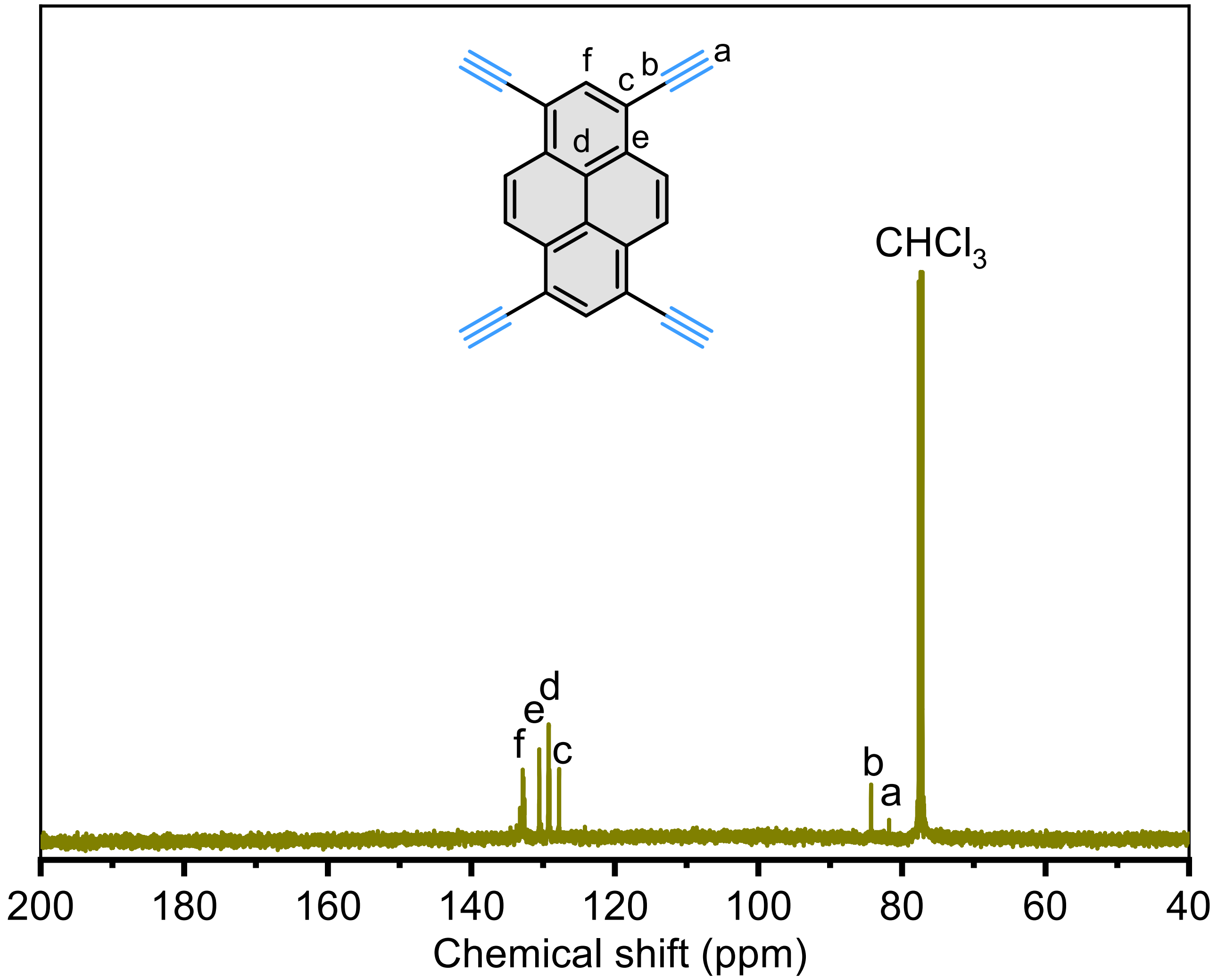

Figure S3. The ^{13}C-NMR spectrum of PyT in $CDCl_3$.

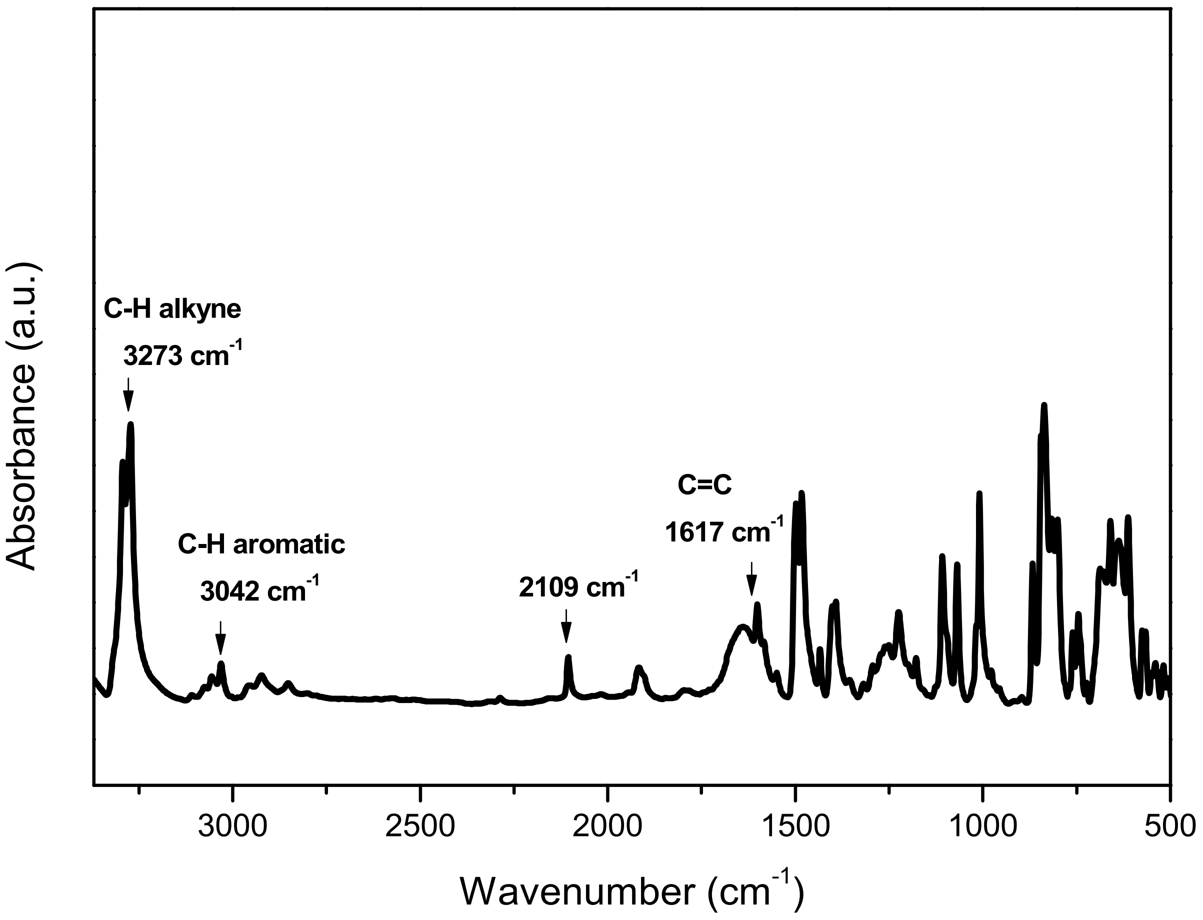

Figure S4. FT-IR spectrum of TPET.

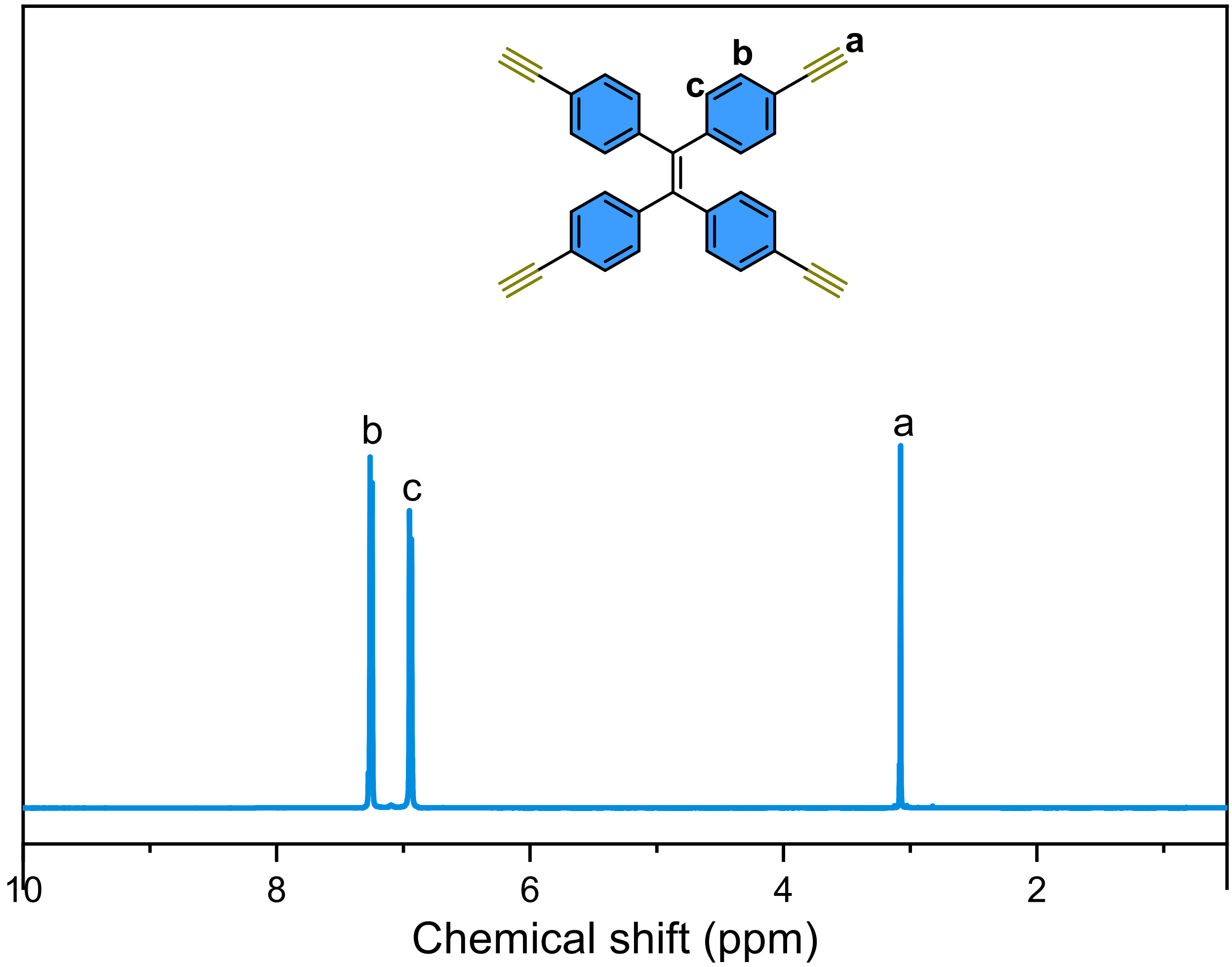

Figure S5. ^{1}H NMR spectrum of TPET in $CDCl_3$.

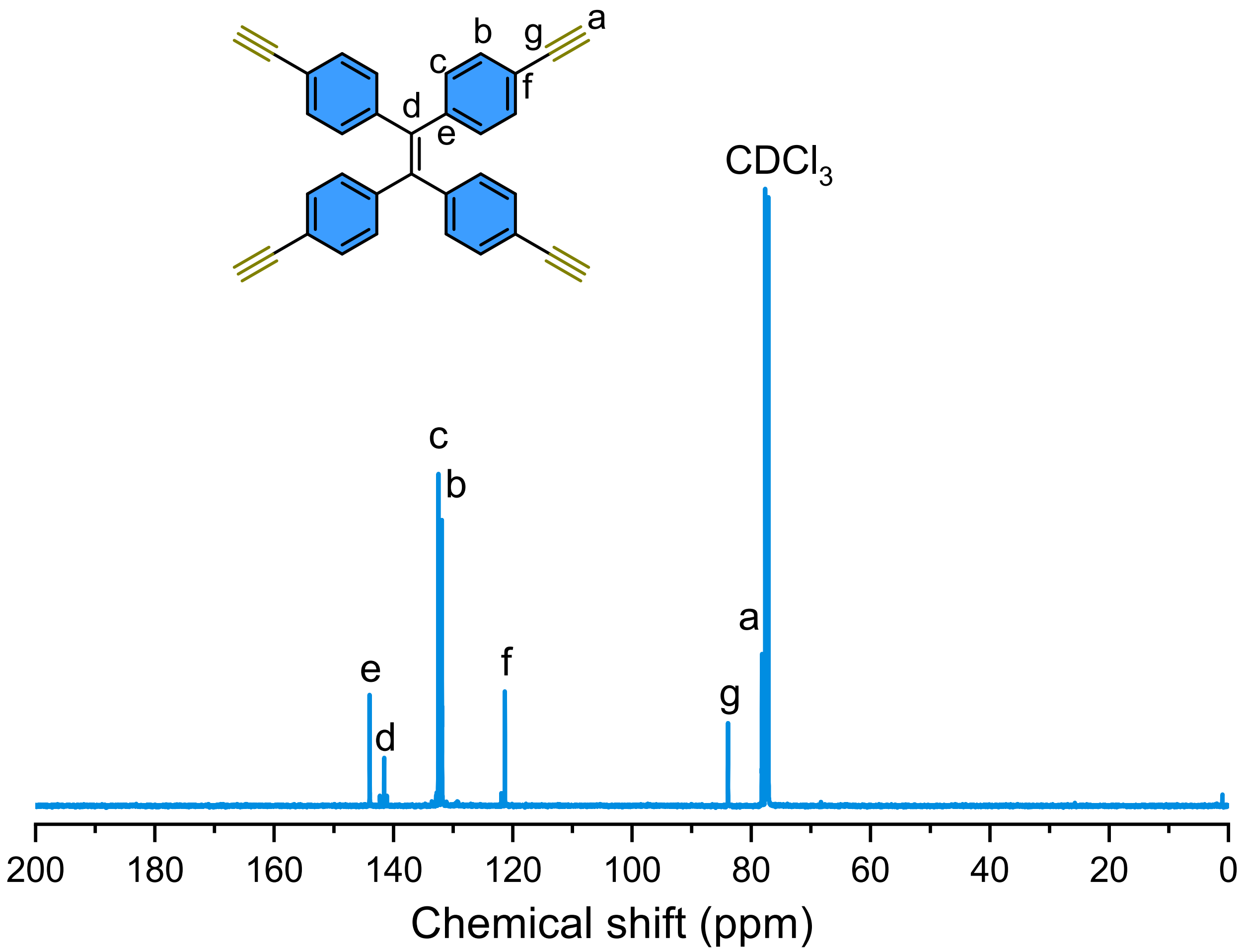

Figure S6. ^{13}C NMR spectrum of TPET in $CDCl_3$.

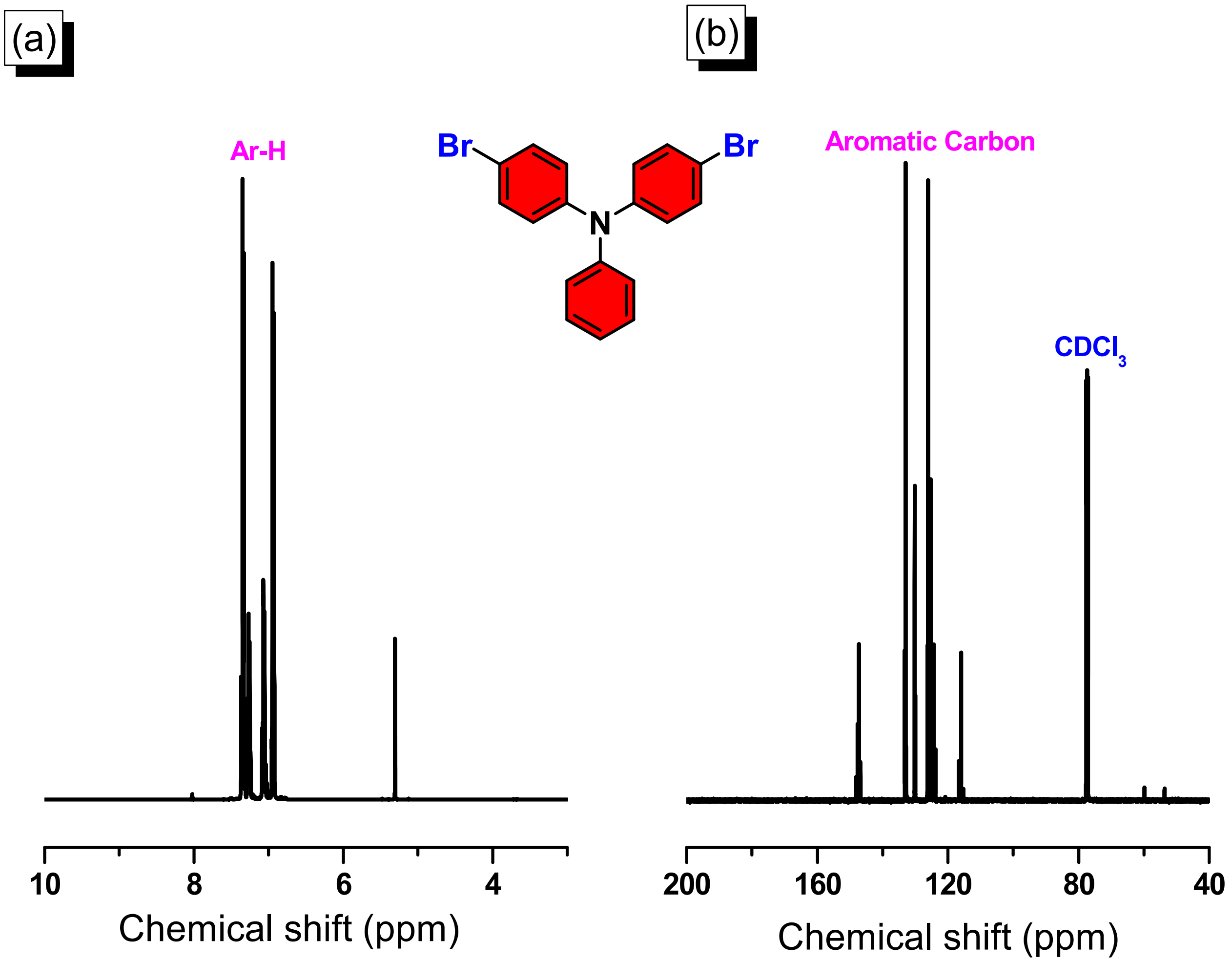

Figure S7. (a) ^{1}H and (b) ^{13}C-NMR spectra of TPA-Br_2

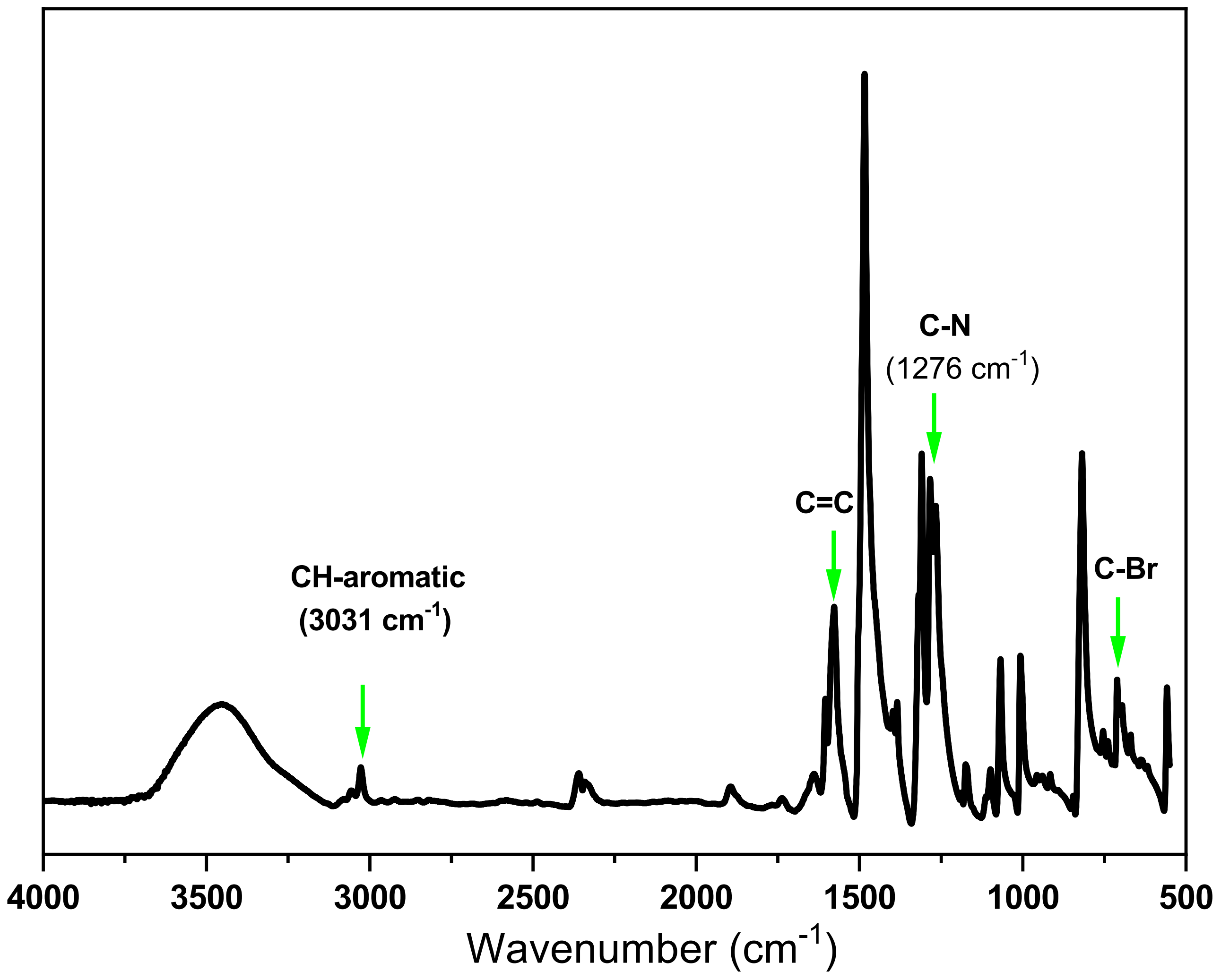

Figure S8. FTIR profile of TPBZ-Br_4.

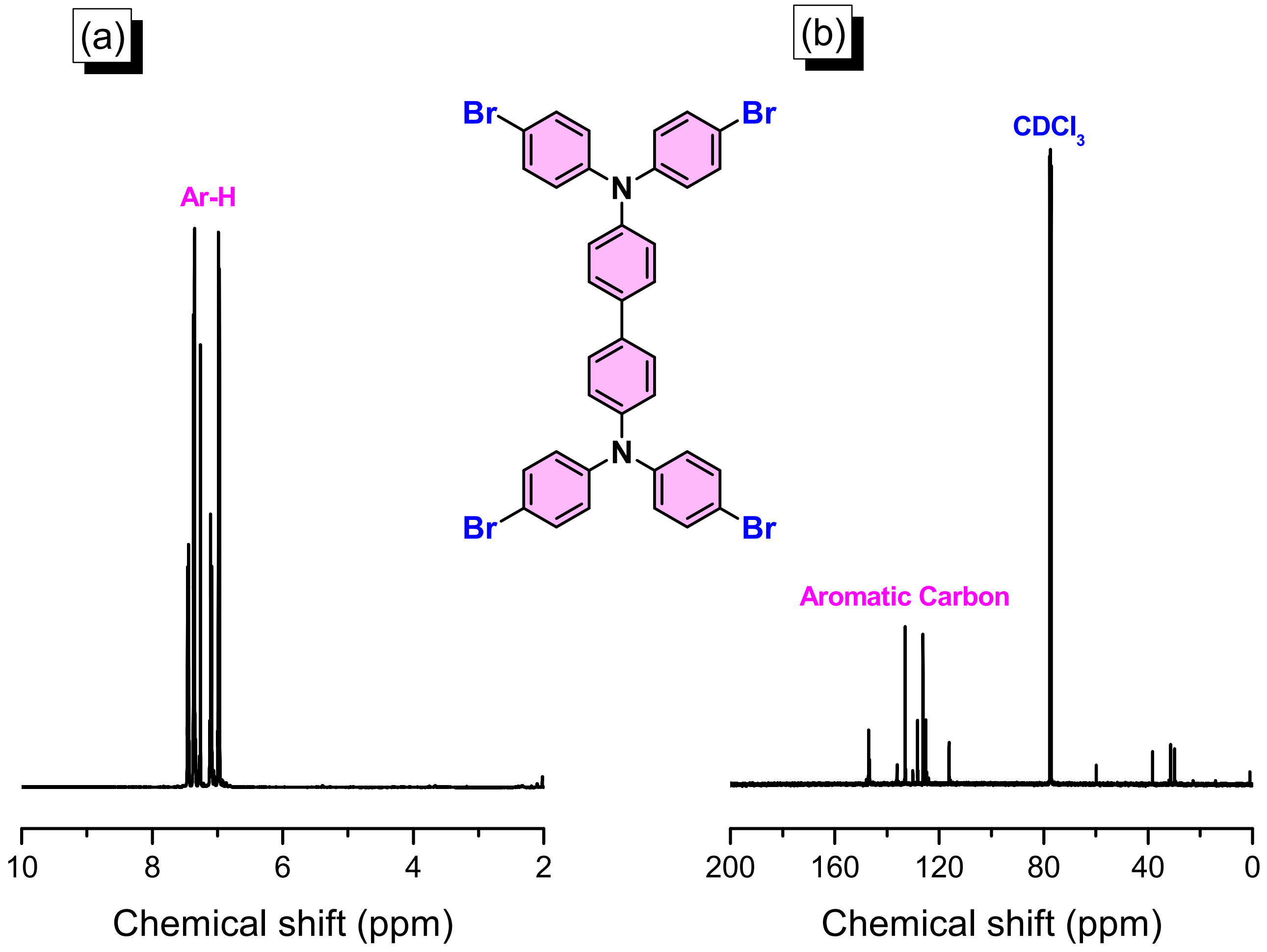

Figure S9. (a) ^{1}H and (b) ^{13}C-NMR spectra of TPBZ-Br_4.

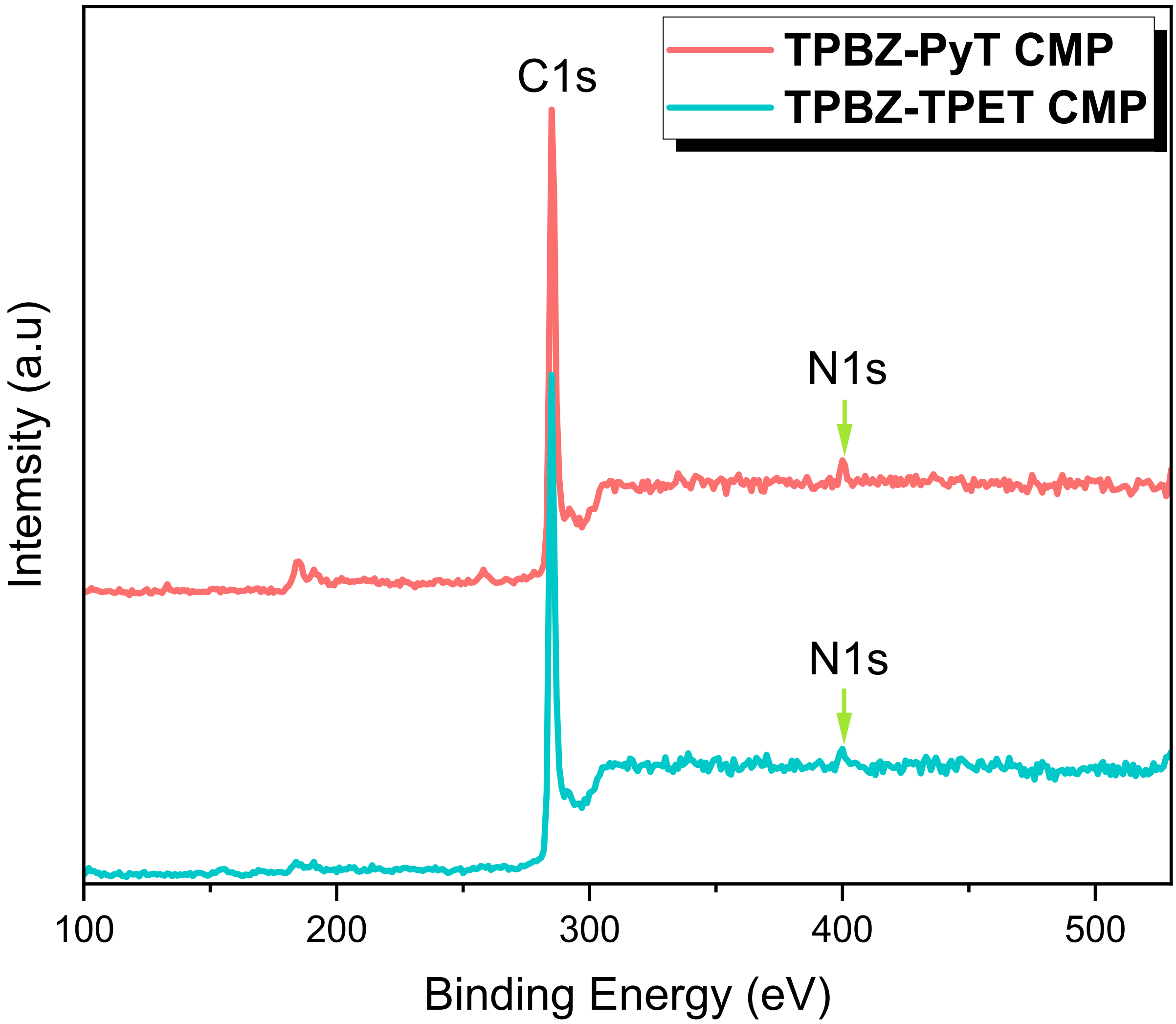

Figure S10. XPS survey profiles of TPBZ-PyT and TPBZ-TPET CMPs.

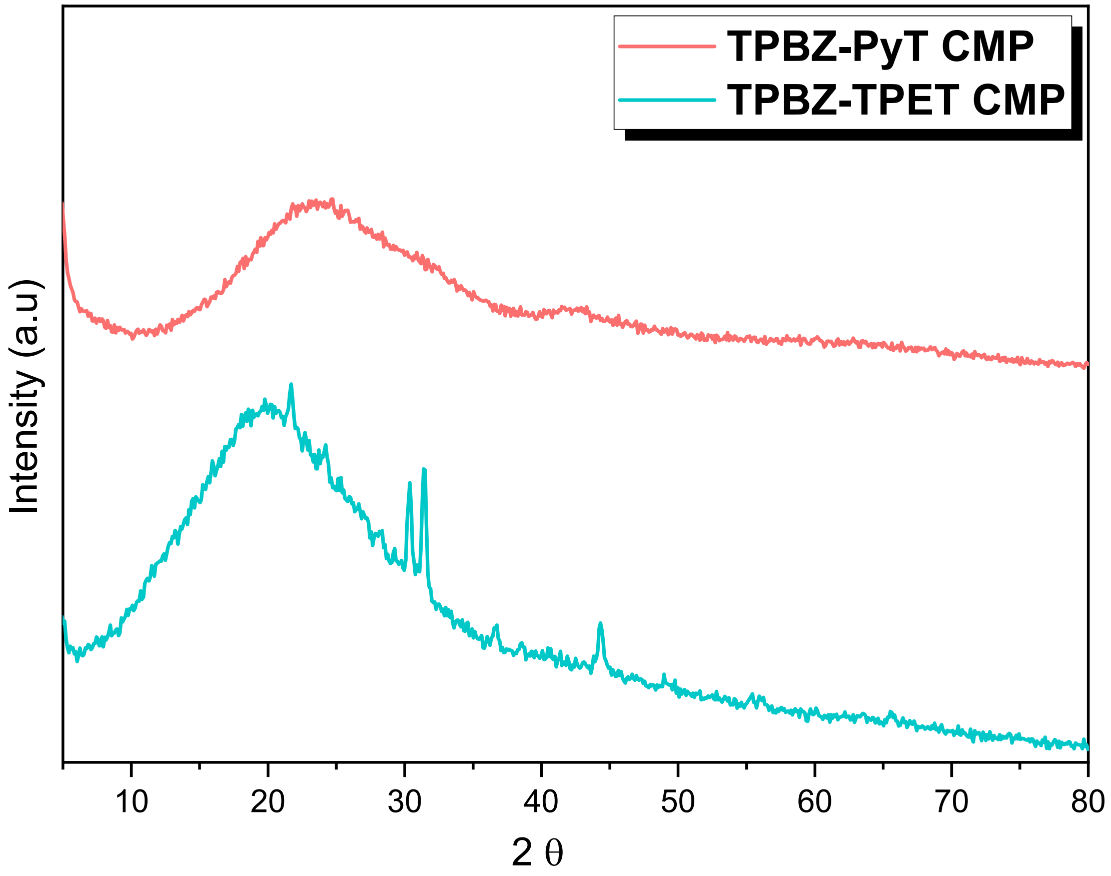

Figure S11. XRD of TPBZ-PyT and TPBZ-TPET CMPs.

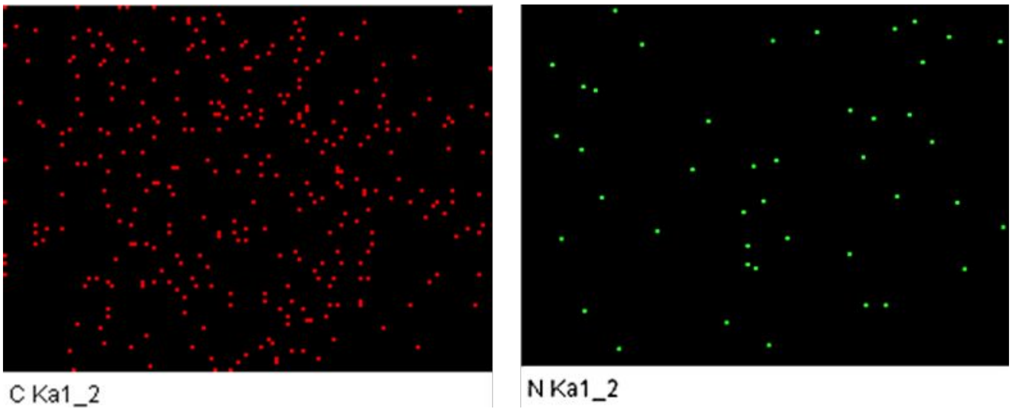

Figure S12. SEM-EDS images for C and N of TPBZ-PyT CMP.

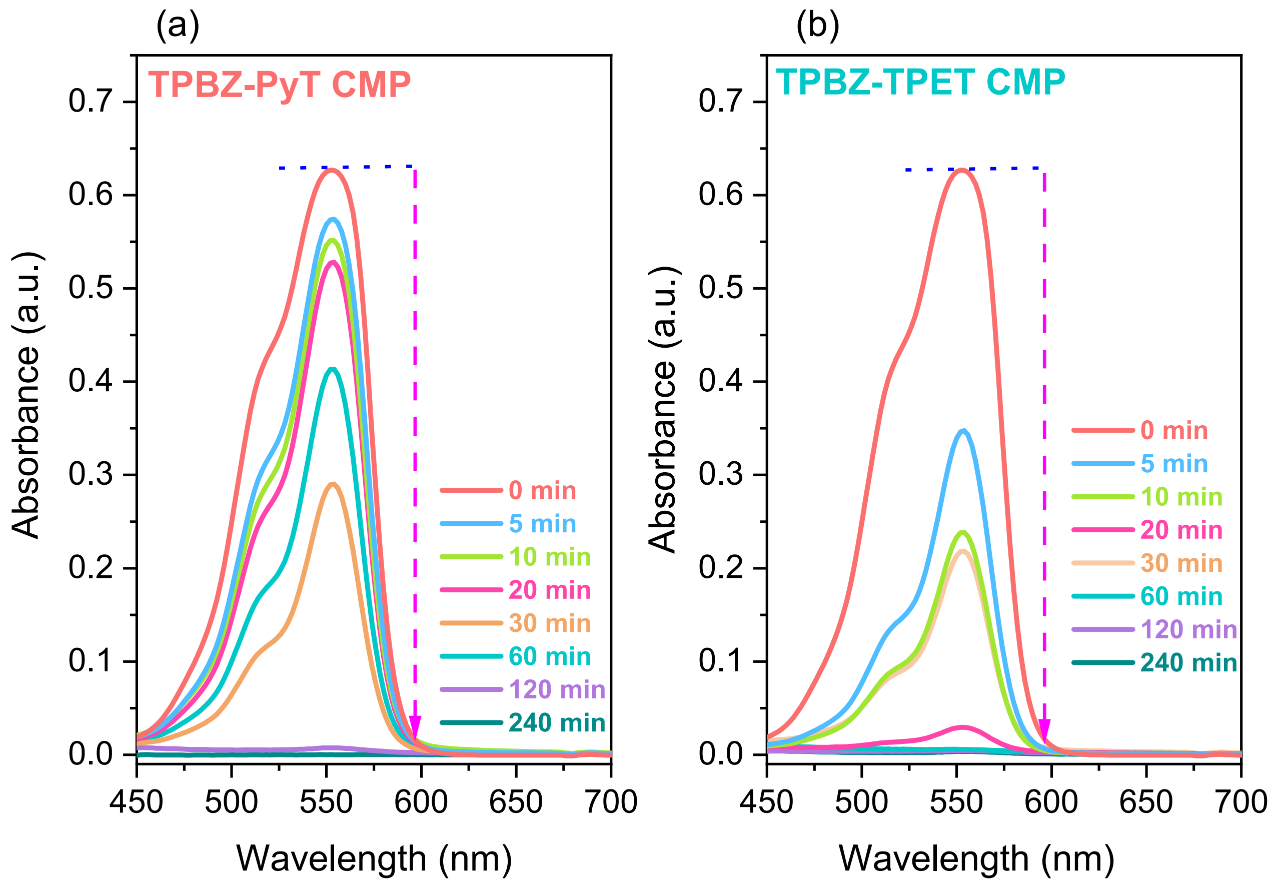

Figure S13. The effect of the contact time on the adsorption properties of (a) TPBZ-PyT and (b) TPBZ-TPET CMPs.

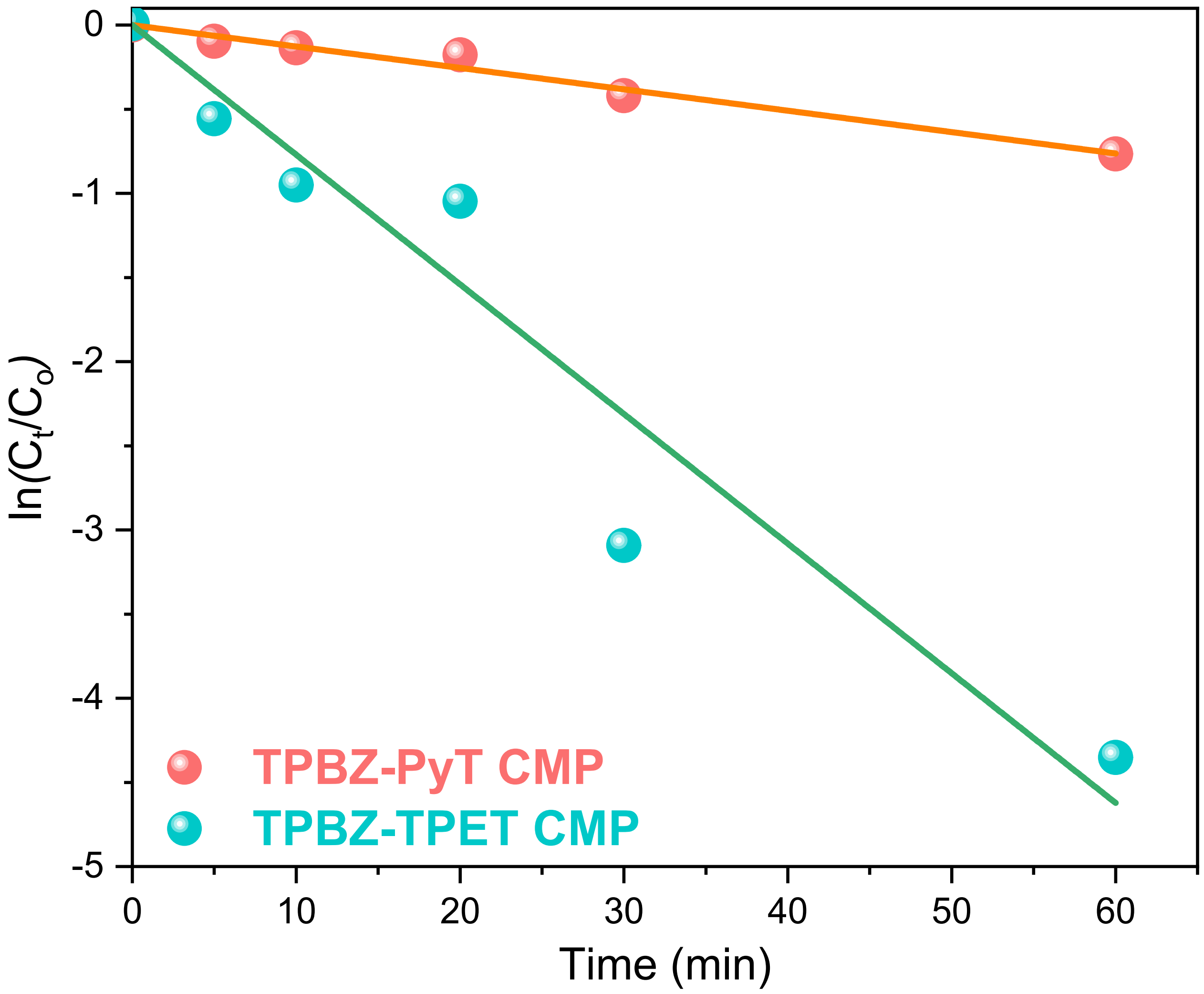

Figure S14. Plots of ln(C_t/C_0) against the reaction time for reducing RhB dyestuff by TPBZ-PyT and TPBZ-TPET CMPs.

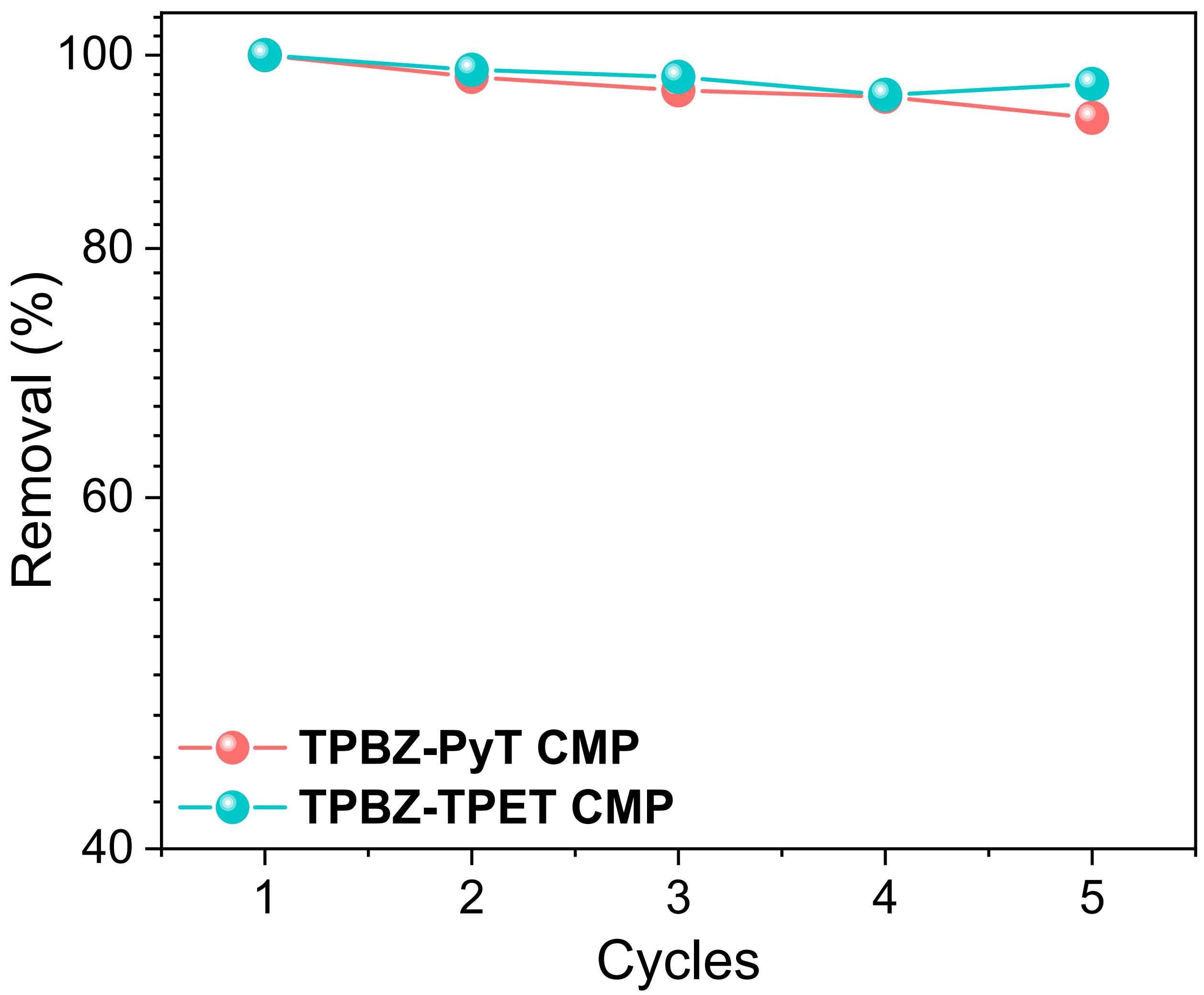

Figure S15. Recycling Profile of TPBZ-PyT and TPBZ-TPET CMPs toward RhB dyestuff adsorption.

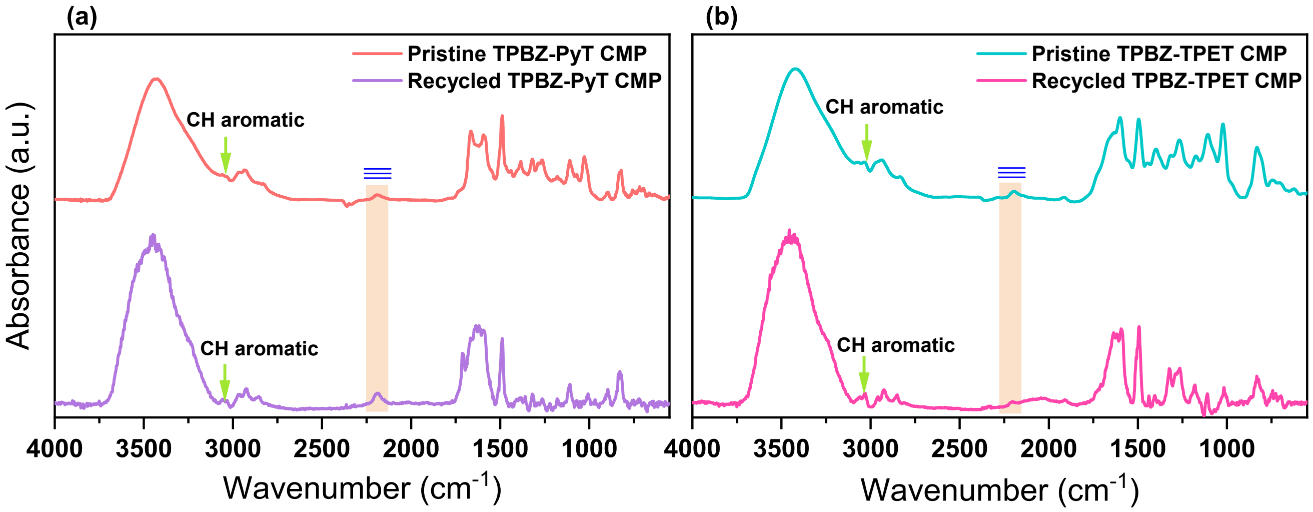

Figure S16. FTIR profiles of (a) pristine and recycled TPBZ-PyT and (b) TPBZ-TPET CMPs.

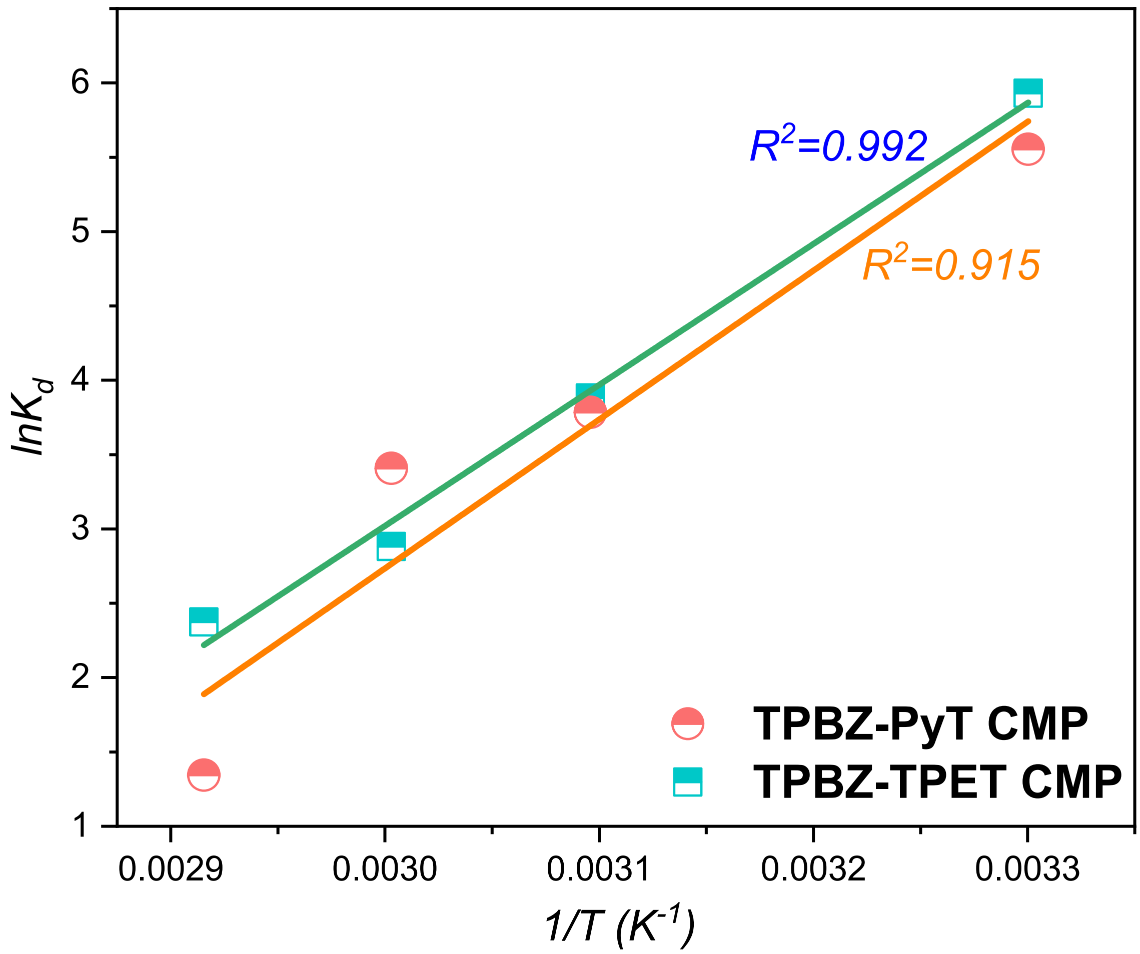

Figure S17. Van't Hoff plots for RhB adsorption onto TPBZ-PyT and TPBZ-TPET CMPs.

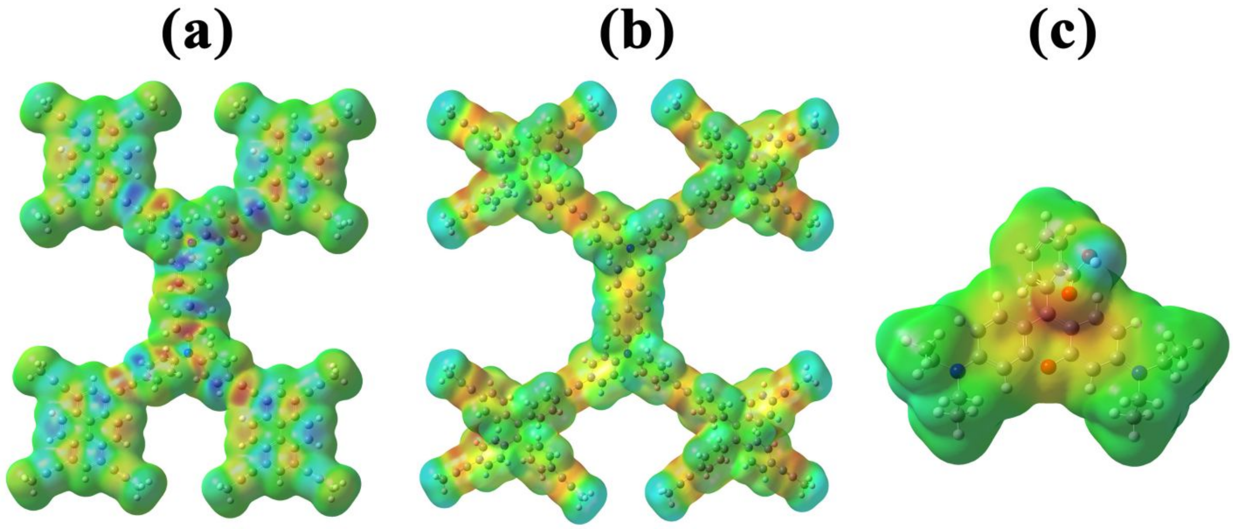

Figure S18. Electrostatic potential (ESP) distributions of (a) TPBZ-PyT CMP, (b) TPBZ-TPET CMP, and (c) RhB dye.

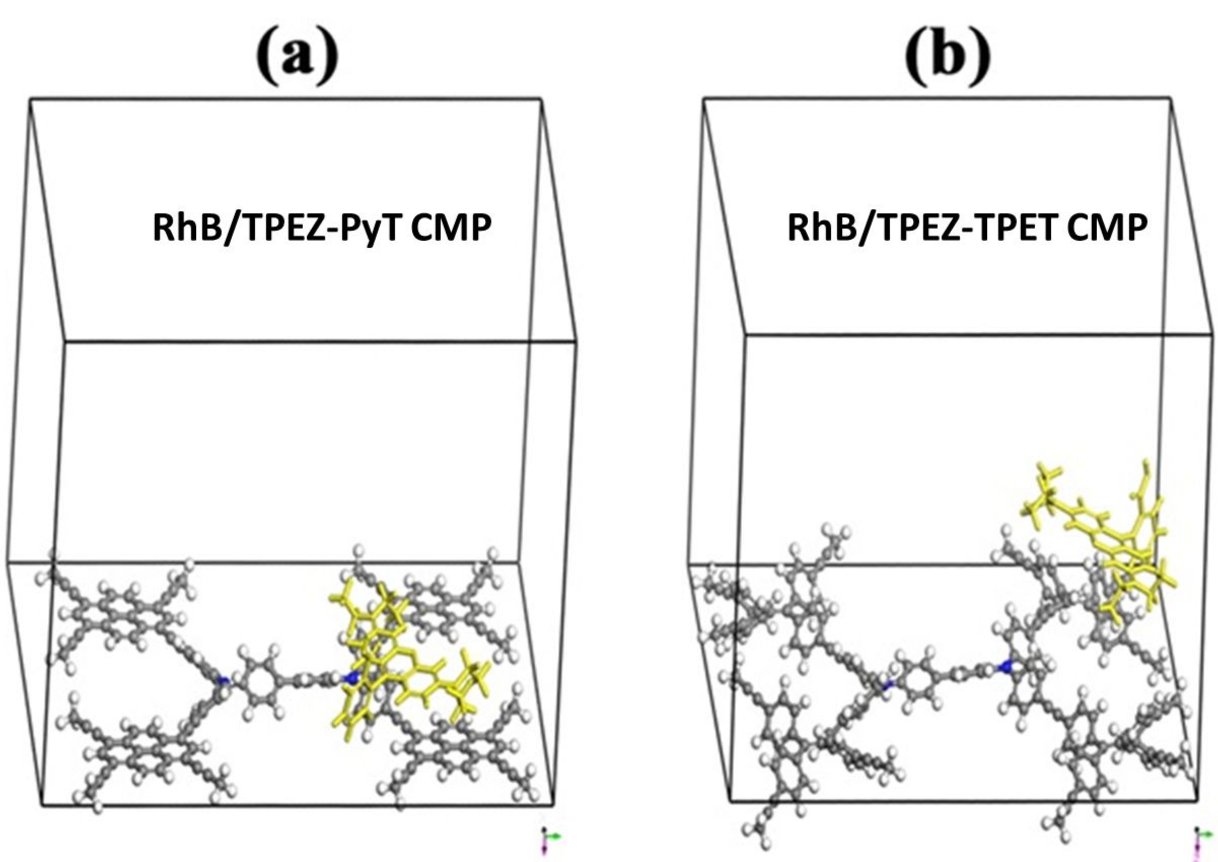

Figure S19. The most stable configuration of (a) RhB dye adsorption over TPBZ-PyT CMP and (b) RhB dye adsorption over TPBZ-TPET CMP.

Table S1. XPS fitting parameters setting of TPBZ-TPET and TPBZ-PyT CMPs.

Status	CMP	TPBZ-PyT		TPBZ-TPET	
	B. E	FWHM	Area	FWHM	Area
**C1s**					
**C=C**	283.85	1.00	6260.83	1.07	7226.94
**C-N**	284.85	0.86	1780.83	0.74	1007.55
**C-O**	285.33	2.24	1360.84	2.12	1254.99
**N1s**					
**N-C**	398.91	1.79	794.26	1.98	351.43

Table S2. Kinetic parameters of pseudo-first-order, pseudo-second-order, and intraparticle diffusion kinetics models.

Samples	Pseudo-first order			Pseudo-second order			Intraparticle diffusion		
	Q_e (mg g^{-1})	k_1 (min^{-1})	R^2	Q_e (mg g^{-1})	k_2 (g mg^{-1} min^{-1})	R^2	K_i	C	R^2
TPBZ-PyT CMP	41.94	0.012	0.97	103.51	4.95×10^{-5}	0.41	4.64	6.27	0.93
TPBZ-TPET CMP	48.36	0.085	0.95	55.55	2.54×10^{-3}	0.99	4.45	11.66	0.78

Table S3. Isotherm parameters of Langmuir and Freundlich isotherms models.

Samples	Langmuir isotherm				Freundlich isotherm			
	Q_{max} (mg L^{-1})	K_L (L g^{-1})	Intercept	R^2	K_F (L$^{1/n}$ mg$^{1/n-1}$ g^{-1})	N	Intercept	R^2
**TPBZ-PyT CMP**	421.94	0.22	0.010	0.991	132.82	3.78	4.88	0.74
**TPBZ-TPET CMP**	704.22	0.98	0.001	0.996	280.05	3.478	5.63	0.85

Table S4. Comparative study between TPBZ-PyT and TPBZ-TPET CMPs with earlier reported porous materials.

**Adsorbents**	Q_m **(mg g^{-1})**	**Ref.**
M3-OBDCA	50.32	[1]
S@TP biochar	32.81	[2]
CMP-YA	535.00	[3]
MSAB	229.60	[4]
PFCMP-0	629.10	[5]
chitosan hydrogel	556.90	[6]
covalent organic polymer	131.23	[7]
graphene hydrogel	341.00	[8]
Noria-POP-1	855.00	[9]
Graphene sponge	72.00	[10]
DAPS-TP-POP	59.20	[11]
CZIF-867	116.20	[12]
AC	32.47	[13]
ZIF-67 loaded ACs-based pellets	47.24	[13]
Sugar-based ACS	123.46	[14]
APEADA	23.30	[15]
CoOF	72.15	[16]
SA-MMNPs	216.0	[17]
**TPBZ-PyT CMP**	**421.94**	**This Study**
**TPBZ-TPET CMP**	**704.22**	

Table S5. The calculated MOs energies, energy gap, and global reactivity parameters of the studied molecules.

Quantum descriptors	TPBZ-PyT CMP	TPBZ-TPET CMP
**E_{HOMO} (eV)**	-4.68	-4.69
**E_{LUMO} (eV)**	-2.15	-1.87
**ΔE (eV)**	2.53	2.82
**Electron affinity (eV)**	2.15	1.87
**Ionization potential (eV)**	4.68	4.69
**Chemical hardness (eV)**	1.27	1.41
**Softness (eV^{-1})**	0.39	0.35
**Electronegativity (eV)**	3.42	3.28
**Electrophilicity index (eV)**	4.61	3.81
**Chemical potential (eV)**	-3.42	-3.28

Table S6. Fukui index of RhB dye calculated by DFT.

Atom Site	Mulliken Charge	Electrophilic Attack (f^-)	Nucleophilic Attack (f^+)
O(1)	-0.446	0.030	0.024
O(2)	-0.439	0.018	0.022
O(3)	-0.409	0.012	0.027
N(4)	-0.381	0.025	0.018
N(5)	-0.380	0.025	0.018
C(6)	0.008	0.062	0.074
C(7)	0.003	0.002	-0.012
C(8)	-0.015	0.002	-0.011
C(9)	0.272	0.019	0.018
C(10)	0.275	0.018	0.017
C(11)	0.209	0.026	0.028
C(12)	0.209	0.026	0.029
C(13)	-0.008	-0.009	0.001
C(14)	-0.159	0.014	0.013
C(15)	-0.160	0.015	0.014
C(16)	-0.099	0.020	0.020
C(17)	-0.097	0.023	0.026
C(18)	-0.123	0.018	0.012

C(19)	-0.125	0.019	0.013
C(20)	-0.032	-0.018	-0.016
C(21)	-0.031	-0.019	-0.017
C(22)	-0.031	-0.019	-0.017
C(23)	-0.032	-0.018	-0.016
C(24)	-0.080	0.014	0.020
C(25)	-0.065	0.000	-0.001
C(26)	-0.203	-0.007	-0.007
C(27)	-0.205	-0.007	-0.006
C(28)	-0.205	-0.007	-0.007
C(29)	-0.202	-0.007	-0.007
C(30)	-0.046	0.009	0.010
C(31)	-0.064	0.016	0.025
C(32)	-0.080	0.020	0.024
C(33)	0.459	0.009	0.020

Table S7. MC simulations calculated outputs and descriptors (kcal/mol) for the most stable adsorption configurations of RhB dye over the surface of the prepared molecules.

Structures	Total energy	Adsorption energy	Rigid adsorption energy	Deformation energy	RhB : dEad/dNi
RhB/TPBZ-TPET CMP	55.35	-79.70	-31.01	-48.69	-79.70
RhB/TPBZ-PyT CMP	56.23	-78.83	-30.70	-48.13	-78.83

References

[1] Zhao, Y.; Yang, H.; Sun, J.; Zhang, Y.; Xia, S. Enhanced Adsorption of Rhodamine B on Modified Oil-Based Drill Cutting Ash: Characterization, Adsorption Kinetics, and Adsorption Isotherm. *ACS Omega*, **2021**, *6*, 17086-17094, https://doi.org/10.1021/acsomega.1c02214.

[2] Vigneshwaran, S.; Sirajudheen, P.; Karthikeyan, P.; Meenakshi, S. Fabrication of sulfur-doped biochar derived from tapioca peel waste with superior adsorption performance for the removal of

Malachite green and Rhodamine B dyes. *Surf. Interfaces*, **2021**, *23*, 100920. doi.org/10.1016/j.surfin.2020.100920.

[3] Yuan, Y.; Huang, H.; Chen, L.; Chen, Y. N, N′-Bicarbazole: a versatile building block toward the construction of conjugated porous polymers for CO_2 capture and dyes adsorption. *Macromolecules*, **2017**, *50*, 4993-5003. doi.org/10.1021/acs.macromol.7b00971.

[4] Joshiba, G. J.; Kumar, P. S.; Govarthanan, M.; Ngueagni, P. T.; Abilarasu, A. Investigation of magnetic silica nanocomposite immobilized Pseudomonas fluorescens as a biosorbent for the effective sequestration of Rhodamine B from aqueous systems. *Environ. Pollut.* **2021**, *269*, 116173. doi.org/10.1016/j.envpol.2020.116173.

[5] Yang, R. X.; Wang, T. T.; Deng, W. Q. Extraordinary capability for water treatment achieved by a perfluorous conjugated microporous polymer. *Sci. Rep.* **2015**, *5*, 10155. doi.org/10.1038/srep10155.

[6] Y. Tang, T. He, Y. Liu, B. Zhou, R. Yang, L. Zhu, Sorption behavior of methylene blue and rhodamine B mixed dyes onto chitosan graft poly (acrylic acid-co-2-acrylamide-2-methyl propane sulfonic acid) hydrogel, Adv. Polym. Tech., 37 (2018) 2568-2578, https://doi.org/10.1002/adv.21932.

[7] Shakeri, S.; Rafiee, Z.; Dashtian, K. Fe_3O_4-based melamine-rich covalent organic polymer for simultaneous removal of auramine O and rhodamine B. *J. Chem. Eng. Data* **2020**, *65*, 696-705. doi.org/10.1021/acs.jced.9b00945.

[8] Zhao, Y.; Zhang, Y.; Liu, A.; Wei, Z.; Liu, S.; Construction of three-dimensional hemin-functionalized graphene hydrogel with high mechanical stability and adsorption capacity for enhancing photodegradation of methylene blue. *ACS Appl. Mater. Interfaces*, **2017**, *9*, 4006-4014. doi.org/10.1021/acsami.6b10959.

[9] Jiang, D.; Deng, R.; Li, G.; Zheng, G.; Guo, H. Constructing an ultra-adsorbent based on the porous organic molecules of noria for the highly efficient adsorption of cationic dyes. *RSC Adv.* **2020**, *10*, 6185-6191. doi.org/10.1039/C9RA08490H.

[10] Zhao, J.; Ren, W.; Cheng, H. M. Graphene sponge for efficient and repeatable adsorption and desorption of water contaminations. *J. Mater. Chem.* **2012**, *22*, 20197-20202. doi.org/10.1039/C2JM34128J.

[11] Du, X. C.; Zhu, J. H.; Quan, Z. J.; Wang, X. C. Adsorption of rhodamine B by organic porous materials rich in nitrogen, oxygen, and sulfur heteroatoms. *New J. Chem.* **2021**, *45*, 3448-3453. doi.org/10.1039/D0NJ05750A.

[12] Zhang, J.; Yan, X.; Hu, X.; Feng, R.; Zhou, M. Direct carbonization of Zn/Co zeolitic imidazolate frameworks for efficient adsorption of Rhodamine B. *Chem. Eng. J.* **2018**, *347*, 640-647.doi.org/10.1016/j.cej.2018.04.132.

[13] Li, Y.; Yan, X.; Hu, X.; Feng, R.; Zhou, M. Trace pyrolyzed ZIF-67 loaded activated carbon pellets for enhanced adsorption and catalytic degradation of Rhodamine B in water. *Chem. Eng. J.* **2019**, *375*, 122003. doi.org/10.1016/j.cej.2019.122003.

[14] Xiao, W.; Garba, Z. N.; Sun, S.; Lawan, I.; Wang, L.; Lin, M.; Yuan, Z. Preparation and evaluation of an effective activated carbon from white sugar for the adsorption of rhodamine B dye. *J. Clean. Prod.* **2020**, *253*,119989. doi.org/10.1016/j.jclepro.2020.119989.

[15] Shen, X.; Ma, S.; Xia, H.; Shi, Z.; Mu, Y.; Liu, X. Cationic porous organic polymers as an excellent platform for highly efficient removal of pollutants from water. *J. Mater. Chem. A*, **2018**, *6*, 20653-20658. doi.org/10.1039/C8TA09145E.

[16] Waheed, A.; Mansha, M.; Kazi, I. W.; Ullah, N. Synthesis of a novel 3, 5-diacrylamidobenzoic acid based hyper-cross-linked resin for the efficient adsorption of Congo Red and Rhodamine B. *J. Hazard. Mater.* **2019**, *369*, 528-538. doi.org/10.1016/j.jhazmat.2019.02.058.

[17] Barylak, M.; Cendrowski, K.; Mijowska, E. Application of carbonized metal–organic framework as efficient adsorbent of cationic dye. *Ind. Eng. Chem. Res.* **2018**, *57*, 4867-4879. doi.org/10.1021/acs.iecr.7b03790.
